# Synthesis of molecular data on Mactroidea (Bivalvia): phylogenetic implications and open questions

**DOI:** 10.7717/peerj.21681

**Published:** 2026-09-30

**Authors:** Ava Ghezelayagh, Stewart M. Edie, Katie S. Collins, Javier H. Signorelli, David Jablonski

**Affiliations:** 1 Department of the Geophysical Sciences, University of Chicago, Chicago, IL, United States of America; 2 Data Science Institute, University of Chicago, Chicago, IL, United States of America; 3 Department of Paleobiology, National Museum of Natural History, Smithsonian Institution, Washington, DC, United States of America; 4 The Natural History Museum, London, United Kingdom; 5 Instituto de Biología de Organismos Marinos (IBIOMAR, CCT CONICET CENPAT), Puerto Madryn, Chubut, Argentina

**Keywords:** Mollusca, Phylogeny, Paleontology, Biogeography, Budding cladogenesis, Systematics, Trough shells, Duck clams, Mesodesmatidae, Anatinellidae

## Abstract

Molecular phylogenetics have clarified high-level evolutionary relationships across the tree of life, but sampling is highly uneven at lower levels, particularly for marine organisms. Here, we synthesize all publicly available Sanger sequencing data for the diverse yet under-sampled marine bivalve superfamily Mactroidea to provide a unified molecular and paleontological framework, and to guide targeted sampling for a more complete understanding of the group’s evolutionary history. We infer two complementary phylogenetic estimates, one using a comprehensive eight-gene alignment that maximizes taxon sampling, and a second high-coverage three-gene alignment that maximizes molecular coverage. Both phylogenies are largely concordant with morphology-based classifications at the genus and subgenus levels, but reveal that the recognized mactroid families with sequence data, as currently described, and the subfamilies of Mactridae are not reciprocally monophyletic. Among the genera, evidence suggests that *Spisulona* and *Taria*, often synonymized to various extant taxa, may be valid genera or subgenera, although further molecular data are needed to confirm these taxonomic implications. Budding-informed time calibration using 18 fossil constraints reveals several conflicts between fossil ages and the inferred molecular topology, likely owing to incomplete fossil and molecular sampling and ambiguity of certain fossil assignments. By tabulating conflicting or missing data, we identify priorities for future molecular sampling, and show that gaps in the molecular data are unevenly distributed geographically, with concentrations in the tropical East Pacific and tropical West Pacific. Combined phylogenetic, biogeographic, and paleontological inventories, supported by voucher-based verification of specimens, can guide targeted sampling to efficiently improve understanding of within-clade relationships and evolutionary dynamics in Mactroidea.

## Introduction

The wide availability of molecular data has yielded many insights into phylogenetic relationships across the Tree of Life. However, for many clades the data are unevenly distributed, so that analyses specifically designed to fill in gaps and resolve ambiguities would be especially valuable. Here, in a single analytical framework, we synthesize all publicly available Sanger sequencing data from a diverse and economically important clade of marine bivalves, the superfamily Mactroidea within Imparidentia. This group of burrowing, suspension-feeding and deposit-feeding clams occurs mostly on the continental shelf and comprises four families: Mactridae (199 living species with the inclusion of Kymatoxinae, as suggested by the analyses presented here), Mesodesmatidae (32 living species), Cardiliidae (four living species), and Anatinellidae (three species with the exclusion of Kymatoxinae).

Building upon existing molecular studies of this group (Ni et al., 2012; Shen et al., 2014; Ma et al., 2023; Ma et al., 2024; Liza, Stotz & Haye, 2024), we present two phylogenetic estimates, one based on a concatenated alignment comprising the most complete sequence for each species and gene, and a second based on the subset of these genes that have been sequenced for at least 50% of the species sampled (as in Streicher, Schulte & Wiens, 2016). We highlight conflicts and commonalities between these two approaches, and briefly explore the phylogenetic and taxonomic implications of this synthesis. We identify open questions, including a proposed list of key targets for future sequencing that could substantially improve our understanding of the group’s phylogenetic structure and evolutionary history.

## Materials and Methods

Using the *taxise* function in the PhyLoTaR pipeline (Bennett et al., 2018), we retrieved National Center for Biotechnology Information (NCBI)’s unique taxonomic identifiers for all daughter taxa of Mactroidea with sequences stored in a local copy of the NCBI GenBank nucleotide database (downloaded June 2025). We used a custom Perl script to extract names and authorities for species in this list, which we cross-referenced with our own inventory of mactroid taxonomy based on recent literature syntheses (*e.g.*, Huber, 2010; Huber, 2015; Signorelli, 2019). The oversplit taxa, misspellings, or subspecies identified through this effort were then corrected or omittted from the analysis. We treated subgenera as operational genera to remain neutral with respect to competing taxonomic hypotheses; this allows us to better integrate paleontological and neontological literature, with the former commonly using subgenera as discrete analytical units. We retrieved all available sequences corresponding to our final list of mactroid species and two outgroups (*Arctica islandica* and *Macridiscus aequilatera*) using Entrez Direct (Sayers, 2010), and relabeled accessions according to our operational taxonomy.

**Figure 1:**
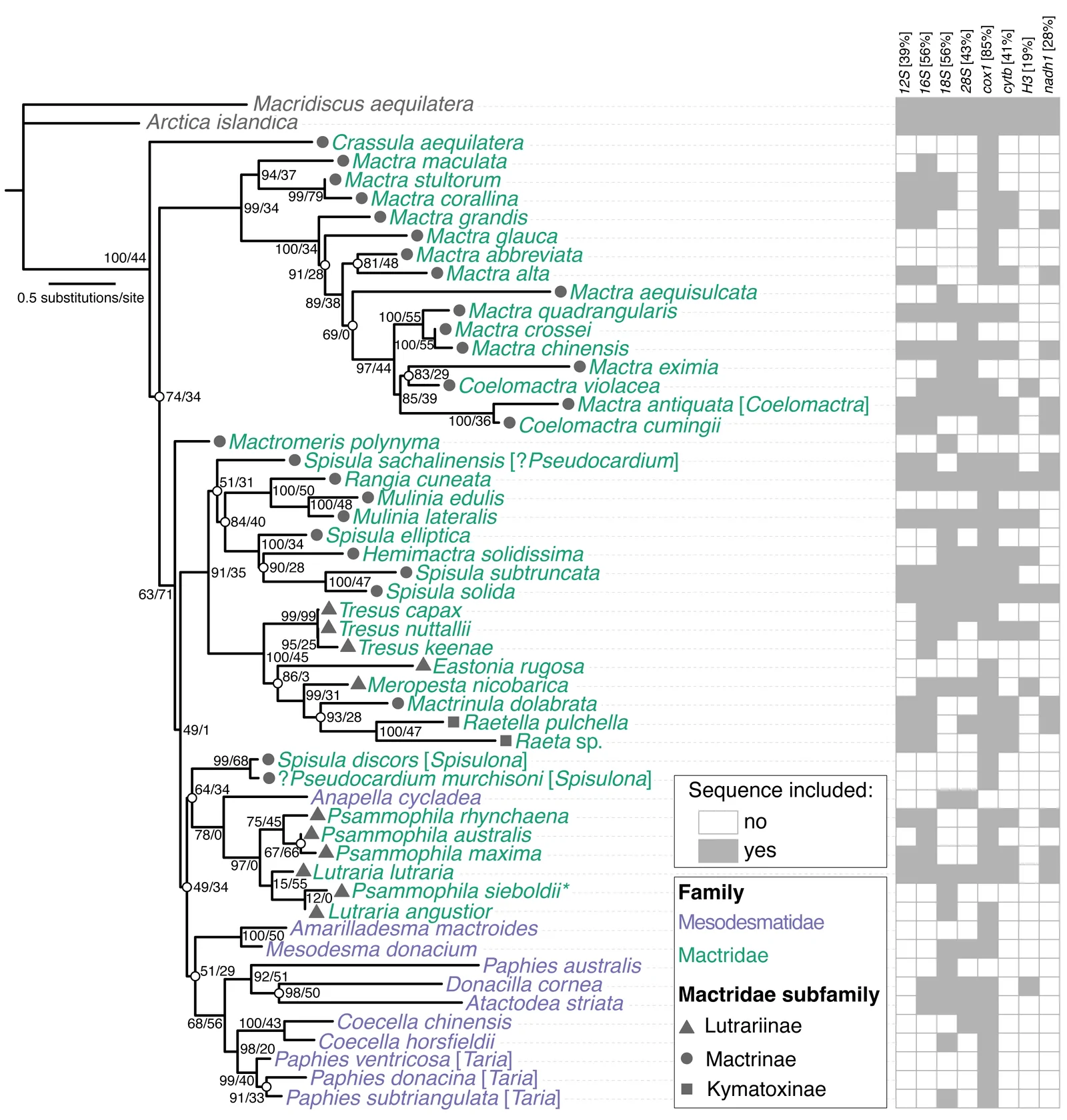
Molecular phylogeny for Mactroidea based on the comprehensive, eight-gene alignment, with inventory of genes sequenced for each species. Percentages beside gene names represent the proportion of taxa with sequence data available for the genes. Values at nodes represent ultrafast bootstrap support (ufBSS)/site concordance factor (sCF) estimates for the subtending branch. White circles at nodes indicate branches with SH-aLRT support values <80%, denoting poor likelihood-based support. Tips are colored by taxonomic family (with Kymatoxinae treated as a subfamily of Mactridae rather than Anatinellidae) and suggested genus assignments follow species names in square brackets. *Psammophila sieboldii* is flagged with an asterisk to denote its alternative placements in the majority-rule consensus tree. The outgroups *Macridiscus aequilatera* (Veneridae) and *Arctica islandica* (Arcticidae) are presented at the top of the figure.

For each species, we merged all sequence accessions into a single FASTA file, and applied a custom locus search term file to parse accessions using SuperCRUNCH (Portik & Wiens, 2020). For the eight genes that have been sequenced from at least ten mactroid species (*12S* rRNA, *16S* rRNA, *cytochrome b* (*cytb*), *cytochrome c oxidase subunit I* (*cox1*), *NADH dehydrogenase subunit 1* (*nadh1*), *histone H3* (*H3*), *18S* rRNA and *28S* rRNA; Fig. 1), we curated three to five sequences to use as references for similarity filtering under a discontiguous megablast algorithm and trimming in SuperCRUNCH. Reference sequence selection prioritized phylogenetic coverage and length. As there were no accessions for Mactroidea exclusively composed of *nadh1* sequences, we used the following accessions from species in Imparidentia for this gene’s reference file: KP699689.1 (Veneridae: *Macridiscus aequilatera*), JX141480.1 (Semelidae: *Scrobicularia plana*), and MT111565.1 (Cardiidae: *Tridacna squamosina*).

We aligned sequences for each gene with Multiple Alignment using Fast Fourier Transform (MAFFT) (Katoh & Standley, 2013) and removed poorly aligned positions, as determined by dataset-specific thresholds, using the ‘gappyout’ algorithm implemented by trimAL v.1.2.59 (Capella-Gutiérrez, Silla-Martínez & Gabaldón, 2009). Before concatenating the single-gene alignments, we identified non-homologous sequences using Basic Local Alignment Search Tool (BLAST) searches (Altschul et al., 1990) and a custom R script (R Core Team, 2025) that evaluates, for each sequence, the proportion of gaps, the number of contiguous non-gap segments, the length of the longest such segment, and the average pairwise identity to other sequences in the alignment. These efforts suggest that the *cox1* sequences available on GenBank are contaminated for the following mactroid taxa: *Oxyperas elongatum* (GU806090.1, GU806134.1, GU806135.1, HM887965.1), *Paphies australis* (HM888032.1, HM888033.1), and *Mactromeris polynyma* (KF643868.1).

Using SuperCRUNCH, we created an eight-gene concatenated alignment (hereafter referred to as the “comprehensive” alignment) and a second alignment (hereafter referred to as the “high-coverage” alignment) comprising the three genes with sequences for at least 50% of the species in the larger alignment: *16S*, *cox1*, and *18S* (see the digital repository on Zenodo for alignment files; https://doi.org/10.5281/zenodo.21728549). We partitioned the concatenated alignments and used the ModelFinderPlus option (MFP+MERGE) in IQ-TREE3 to identify best-fitting nucleotide substitution models (Kalyaanamoorthy et al., 2017; Wong et al., 2026). The best-fit partition model for the comprehensive analysis was as follows: GTR+F+I+G4 for a partition comprising *12S*, *cytb* codon 1, and *nadh1* codon 1; TIM3+F+I+R4 for *16S* TIM+R3 for a partition comprising *18S* and *H3* codons 1 and 2; TIM3+F+R3 for *28S*; GTR+F+I+G4 for *cox1* codon 1; TVM+F+I+R2 for *cox1* codon 2, *cytb* codon 2, and *nadh1* codon 2; HKY+F+G4 for *cox1* codon 3 and *cytb* codon 3; GTR+I+G4 for *H3* codon 3; and HKY+F+R2 for *nadh1* codon 3. The best-fit partition model for the high-coverage analysis was as follows: GTR+F+R3 for *16S*; TN+I+R2 for *18S*; and TIM2+F+I+G4, TVM+F+G4, and TN+F+R3 for *cox1* codons 1, 2, and 3, respectively.

We then inferred maximum-likelihood phylogenies for each dataset under the selected models in IQ-TREE3, and generated 5,000 ultrafast bootstrap trees (Hoang et al., 2018). We repeated this approach, including the selection of best-fitting models of evolution, to infer gene trees for the eight individual-gene data matrices. These gene trees allowed us to calculate gene concordance factors (gCFs; Minh et al., 2020) for the best-scoring trees from the concatenated analyses, which represent the percentage of decisive genes supporting each internal branch. We also calculated likelihood-based site concordance factors (sCFs; Mo et al., 2023), which represent the percentage of decisive sites supporting each internal branch. We further assessed branch support for the resulting best-scoring trees in IQ-TREE3 using 5,000 Shimodaira-Hasegawa-like approximate likelihood-ratio test (SH-aLRT) replicates (Guindon et al., 2010; see the digital respository on Zenodo for estimates of branch support and individual gene trees; https://doi.org/10.5281/zenodo.21728549).

**Figure 2:**
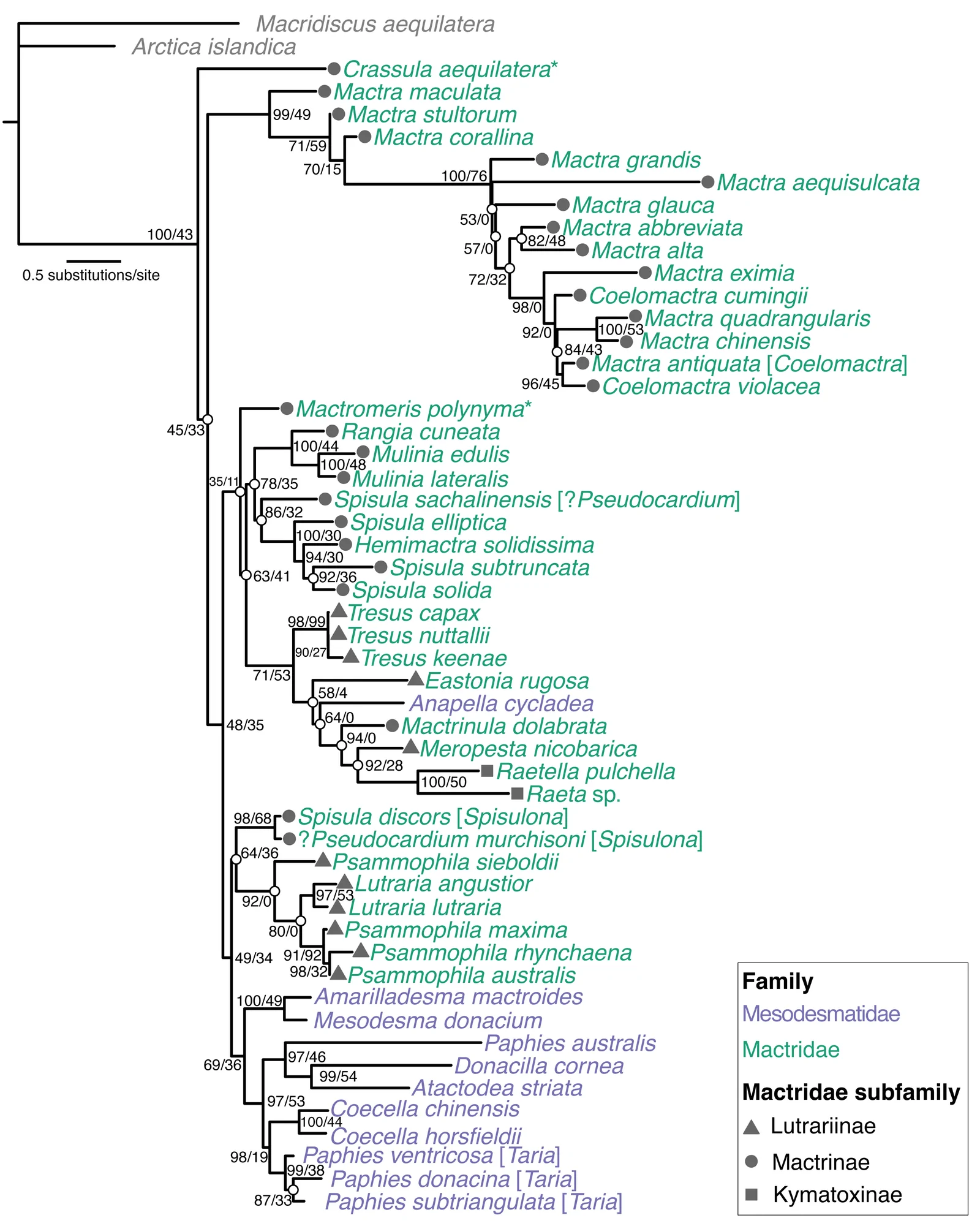
Best-scoring maximum-likelihood molecular phylogeny for Mactroidea, based on the high-coverage alignment comprising the three genes that were sequenced for at least 50% of the species sampled. Values at nodes represent ultrafast bootstrap support (ufBSS)/site concordance factor (sCF) estimates for the subtending branch. White circles at nodes indicate branches with SH-aLRT support values <80%, denoting poor likelihood-based support. Tips are colored by taxonomic family (with Kymatoxinae treated as a subfamily of Mactridae rather than Anatinellidae) and suggested genus assignments follow species names in square brackets. Asterisks denote taxa with alternative placements in the majority-rule consensus tree, which were dropped from the phylogeny before performing the time calibration analyses represented in Fig. 3. The outgroups *Macridiscus aequilatera* (Veneridae) and *Arctica islandica* (Arcticidae) are presented at the top of the figure.

We estimated a time-calibrated genus-level phylogeny by pruning the best-scoring species-level high-coverage tree (Fig. 2) to one representative per genus, using type species of genera where possible. We also pruned *Crassula aequilatera* and *Mactromeris polynyma* from the tree due to their unstable placements (see Results); their inclusion would affect the fossil calibration scheme. As in Crouch et al. (2021), the branch lengths of the pruned phylogeny were scaled to geologic time using treePL (Smith & O’Meara, 2012). Applying a budding model of evolution to our time calibration, 18 nodes were dated by the stratigraphic intervals of the younger of the two lineages branching from each node (as in Crouch et al., 2021); minimum and maximum age constraints to each node were based on the stratigraphic intervals containing the first known appearances of genera. The ages of taxa used for time calibration are provided along with references in Table S1. We specified the “thorough” option to ensure analyses ran until the penalized likelihood converged, and the “randomcv” option set to 1,000 to allow the cross-validation procedure to estimate the optimal smoothing parameter for penalizing rate variation across the phylogeny.

We mapped the known geographic distributions of extant mactroid species using the occurrence data from Huang et al. (2023). We then summarized richness patterns of sequenced and unsequenced species and genera among the “ecoregions” of the Spalding et al. (2007) marine provinces; *Raeta* sp. was not included among the sequenced taxa due to it not being identified to the species level. Occurrence data are provided in Supplemental Dataset S1, in addition to NCBI accession numbers for all sequences and calibrations used for divergence time estimation.

## Results

### Phylogenetic inference

The comprehensive, eight-gene data matrix comprises 7,019 base pairs from 54 mactroid species, with 51% missing data. The high-coverage, three-gene dataset (*16S*, *cox1*, and *18S*) improved matrix occupancy by excluding genes with sparse sampling (3,034 base pairs with 36% missing data), and lacks just one taxon present in the comprehensive alignment (*Mactra crossei*). The phylogenies inferred from these two alignments are mostly congruent, with both trees exhibiting the non-reciprocal monophyly of the Mactridae subfamilies, the nesting of Kymatoxinae and Mesodesmatidae within Mactridae, and the monophyly of Mesodesmatidae, to the exclusion of *Anapella cycladea*. Minor topological differences are restricted to a few internal relationships: among *Mactra* species and the non-monophyletic genus nested within it, *Coelomactra*; the placement of *Spisula* (?*Pseudocardium*) *sachalinensis* relative to the other species of *Spisula*; the positions of *Anapella cycladea* relative to the rest of Mesodesmatidae; the intrarelationships of within *Lutraria* and its subgenus *Psammophila*; and the sister group of Kymatoxinae (*Mactrinula* in the comprehensive tree and *Meropesta* in the high-coverage tree; Figs. 1 and 2).

Despite this general congruence, support for early-diverging mactroid relationships remains low in both the comprehensive and high-coverage trees (Figs. 1 and 2), consistent with dataset heterogeneity, substitutional saturation in faster–evolving mitochondrial genes, and the relatively deep evolutionary timescale for Mactroidea. In addition to low ultrafast bootstrap support, most of the deepest branches in both the comprehensive and high-coverage trees exhibit gene concordance factor (gCF) values of zero, indicating that no gene trees decisively support these relationships. Site concordance factor (sCF) values, representing the percentage of sites supporting the most favored of the three possible resolutions for a branching quartet, are likewise low (<40%) for most of these branches. Low bootstrap support, gCF and sCF values are consistent with the presence of short internal branches deep in the trees (Figs. 1 and 2), and these combined patterns suggest limited phylogenetic signal, potentially reflecting rapid divergence in deep-time and/or incomplete lineage sorting. These patterns may be further complicated by the loss of the many extinct mactroid clades inventoried by Signorelli (2023).

Several “rogue” taxa exhibit unstable placements, with two taxa in Mactrinae (*Crassula aequilatera* and *Mactromeris polynyma*) frequently inferred as early-diverging, now-monospecific lineages. Each represented by just one gene sequence in the concatenated alignments, *Crassula* and *Mactromeris* exhibit inconsistent relationships across analyses, and even between the maximum-likelihood and summarized consensus trees from a single analysis (see asterisks by tip labels in Figs. 1 and 2). Removal of these sequences and realignment does not significantly improve ultrafast bootstrap and site support for the deep branches, and the high-coverage, 3-gene tree shows no topological changes (see tree files in the digital repository on Zenodo). Relationships near the base of the comprehensive, 8-gene supplemental analysis do change with the exclusion of these “rogues” (*e.g.*, Mesodesmatidae becomes polyphyletic and is the earliest-diverging mactroid lineage). However, these relationships are incongruent with the comprehensive and high-coverage trees (Figs. 1 and 2) and are in greater conflict with the fossil record. *Crassula* and *Mactromeris* are among the earliest fossil occurrences of extant mactroids (with *Crassula* dating to the Middle Eocene and *Mactromeris* possibly to the Paleocene but more securely in the Late Eocene (see Table S1)), and so their topological instability is unfortunate. Thus, the likely origin of crown-group mactroids is early Cenozoic, but the molecular topology of extant genera does not match the taxonomy applied to the oldest Cenozoic fossils (*Crassula* and *Mactromeris*, but see also discussion of *Spisula* and *Mulinia* below).

### Taxonomic implications for Mactroidea

#### Families

Mactroidea today comprises four families according to most authorities (Huber, 2015; Signorelli & Carter, 2016; Signorelli, 2019; MolluscaBase, 2025). These are:

**Anatinellidae**: The monogeneric subfamily Anatinellinae, potentially dating back to the Late Miocene (*Anatinella* sp., NMITA web site, accessed Nov 2004), has not been sequenced and so its position and status are unknown. The subfamily Kymatoxinae was reassigned to Anatinellidae from Mactridae by Signorelli & Carter (2016) and MolluscaBase (2025), but is represented in our molecular trees by a well-supported clade comprising *Raeta* and *Raetella* that is firmly nested within Mactridae and sister group to one of two mactrid species: *Mactrinula dolabrata* (Fig. 1, ufBSS = 93%; sCF = 28%) or *Meropesta nicobarica* (Fig. 2, ufBSS = 92%; sCF = 28.1%). The results of Ma et al. (2023), Ma et al. (2024) also support the placement of Kymatoxinae in the family Mactridae, and so we herein refer to the group as Kymatoxinae. Whether this nesting indicates that the expanded Anatinellidae should revert to subfamily status within Mactridae, as in Dall (1903), or the Kymatoxinae should be shifted back into Mactridae with Anatinellidae remaining as a distinct family, is not yet determined.

**Cardiliidae**: This monogeneric family, potentially dating back to the Paleocene (*Cardilia* sp. nov. of Moroz, 1972) has not been sequenced and so its position and status remain unclear (see Table 1). Bieler, Carter & Coan (2010:129) noted that “With shell morphology otherwise suggesting similarity to the Cardiidae, but an internal ligament, this family would be a prime candidate for molecular investigation” (see also Signorelli & Raven, 2018).

**Table 1:** Open questions: potential target species for molecular sequencing. When multiple extant species are assigned to an unsequenced genus, only the type species is listed (T); if the type species is extinct, other extant species are included; (M) = monospecific genus or subgenus today. The first biogeographic region given is for the species listed in the second column, followed by further distribution of additional species in that genus or subgenus, if outside the first-listed region.

**Family**	**Genus/subgenus and species**	**Biogeography**	**Reasoning**
Anatinellidae	*Anatinella nicobarica* (Gmelin, 1791) (T)	Indo-West Pacific	Family (or subfamily, depending on placement of Kymatoxinae) not sequenced (M)
Cardiliidae	*Cardilia semisulcata* (Lamarck, 1819) (T)	Indo-West Pacific, also in West Africa	Family not sequenced
Mactridae	*Anatina anatina* (Spengler, 1802) (T)	West Atlantic, also in East Atlantic, East Pacific	Genus not sequenced
Mactridae	*Austromactra contraria* (Reeve, 1854) (T)	Southern Australia	Genus not sequenced
Mactridae	*Barymactra rostrata* (Spengler, 1802)	East Atlantic	Genus not sequenced; type species extinct (M)
Mactridae	*Crassula aequilatera* (Reeve, 1854)	New Zealand	Unstable placement for genus (rogue taxon) (M)
Mactridae	*Cyclomactra tristis* (Reeve, 1854) (T)	New Zealand	Genus not sequenced
Mactridae	*Darina solenoides* (King, 1832) (T)	Southwest Atlantic	Genus not sequenced
Mactridae	*Diaphoromactra versicolor* (Tate, 1887) (T)	Southern Australia	Genus not sequenced (M)
Mactridae	*Harvella elegans* (Sowerby I, 1825) (T)	East Pacific	Genus not sequenced (M)
Mactridae	*Heterocardia gibbosula* Deshayes, 1855 (T)	Indo-West Pacific	Genus not sequenced (M)
Mactridae	*Huberimactra grayi* (Huber, 2015) (T)	East Atlantic	Genus not sequenced
Mactridae	*Longimactra elongata* (Quoy & Gaimard, 1835) (T)	New Zealand	Genus not sequenced
Mactridae	*Lutraria* (*Goniomactra*) *impar* Reeve, 1854 (T)	Indo-West Pacific	Subgenus not sequenced (M)
Mactridae	*Mactra* (*Maorimactra*) *ordinaria* E. A. Smith, 1898 (T)	New Zealand	Subgenus not sequenced (M)
Mactridae	*Mactra* (*Nannomactra*) *jacksonensis* E. A. Smith, 1885	Southern Australia	Subgenus not sequenced (M)
Mactridae	*Mactrella striatula* (Linnaeus, 1767) (T)	Indo-West Pacific	Genus not sequenced (M)
Mactridae	*Mactrellona alata* (Spengler, 1802) (T)	West Atlantic, also in East Pacific	Genus not sequenced
Mactridae	*Mactromeris polynyma* (Stimpson, 1860) (T)	North Pacific & North Atlantic	Unstable placement for genus (rogue taxon)
Mactridae	*Mactrotoma* (*Electomactra*) *explanata* (Reeve, 1854) (T)	Southern Australia	Subgenus not sequenced (M)
Mactridae	*Mactrotoma* (*Mactroderma*) *velata* (Philippi, 1849) (T)	East Pacific, also in South Australia	Subgenus not sequenced
Mactridae	*Mactrotoma* (*Mactrotoma*) *fragilis* (“Chemnitz” Gmelin, 1791) (T)	West Atlantic, also in East Atlantic, Indo-West Pacific, and East Pacific	Genus not sequenced
Mactridae	*Mactrotoma* (*Micromactra*) *californica* (Conrad, 1837) (T)	East Pacific, also in West Atlantic	Subgenus not sequenced
Mactridae	*Notospisula trigonella* (Lamarck, 1818)	Circum-Australia	Genus not sequenced
Mactridae	*Oxyperas lentiginosum* (Gould, 1852) (T)	Indo-West Pacific, also in East Atlantic	Genus not sequenced; available sequences attributed to *O. elongatum* (GU806090.1, GU806134.1, GU806135.1, and HM887965.1) appear to be contaminated
Mactridae	*Pseudoxyperas aspersum* (Sowerby I, 1825) (T)	Indo-West Pacific	Genus not sequenced (M)
Mactridae	*Raeta* (*Raetina*) *pellicula* (Reeve, 1854)	Indo-West Pacific	Subgenus not sequenced (M)
Mactridae	*Rangia* (*Rangianella*) *mendica* (Gould, 1851) (T)	East Pacific, also in West Atlantic	Subgenus not sequenced
Mactridae	*Resania lanceolata* Gray, 1853 (T)	New Zealand	Genus not sequenced (M)
Mactridae	*Scalpomactra scalpellum* (Reeve, 1854) (T)	New Zealand	Genus not sequenced (M)
Mactridae	*Scissodesma* (*Crepispisula*) *acutissima* (von Cosel, 1995)	East Atlantic	Subgenus not sequenced; type species extinct
Mactridae	*Scissodesma* (*Scissodesma*) *spengleri* (Linnaeus, 1767) (T)	Southern Africa	Subgenus not sequenced (M)
Mactridae	*Simomactra dolabriformis* (Conrad, 1867) (T)	East Pacific, also in Indian Ocean to South Africa	Genus not sequenced
Mactridae	*Trinitasia iheringi* (Dall, 1903)	West Atlantic	Genus not sequenced; type species extinct (M)
Mactridae	*Tumbeziconcha thracioides* (Adams & Reeve, 1850) (T)	East Pacific, also in Indo-West Pacific to South Africa	Genus not sequenced
Mactridae	*Zenatia acinaces* (Quoy & Gaimard, 1835) (T)	New Zealand	Genus not sequenced (M)
Mactridae	*Zenatina victoriae* (Pritchard & Gatliff, 1903) (T)	New Zealand	Genus not sequenced (M)
Mesodesmatidae	*Anapella cycladea* (Lamarck, 1818) (T)	Southern Australia, also possibly in Indo-West Pacific	Anomalous placement at family level
Mesodesmatidae	*Davila plana* (Hanley, 1843) (T)	Indo-West Pacific	Genus not sequenced (M)
Mesodesmatidae	*Mesodesma* (*Ceronia*) *deauratum* (Turton, 1822) (T)	Northwest Atlantic	Subgenus not sequenced
Mesodesmatidae	*Paphies* (*Amesodesma*) *elongata* (Reeve, 1854) (T)	Southern Australia, also in Indo-West Pacific	Subgenus not sequenced
Mesodesmatidae	*Regterenia africana* (Turton, 1932) (T)	West Indian Ocean	Genus not sequenced (M)
Mesodesmatidae	*Soleilletia abbadiana* Bourguignat, 1885	West Indian Ocean	Genus not sequenced (M)

**Mesodesmatidae**: This diverse family may date back to the Late Cretaceous, but the family- and even superfamily-level placements of those extinct genera have been questioned (Saul, 1989). Assuming the Late Cretaceous affiliations are invalid, the family would enter the fossil record in the mid-Eocene (Beu, 2019). The comprehensive and high-coverage trees infer a monophyletic Mesodesmatidae (Figs. 1 and 2; ufBSS/sCF = 51/29% and 69/36%, respectively), to the exclusion of *Anapella*, which is represented by its type species (*A. cycladea*) that is far removed from its confamilials and has unstable placement caused by limited phylogenetic signal (sCF = 0%; Figs. 1 and 2). We find that Mesodesmatidae nests within Mactridae, consistent with the results of previous studies (Taylor et al., 2007; Liza, Stotz & Haye, 2024). The single-gene ML analyses of Liza, Stotz & Haye (2024) resulted in greater polyphyly and paraphyly within the family, extending beyond the placement of *A. cycladea*. In contrast, Taylor et al. (2007) inferred mesodesmatid monophyly (including *A. cycladea*) using a concatenated Bayesian approach, albeit with more limited taxon sampling. Further sequencing within this group will be essential to assess the placement of *Anapella* and define Mesodesmatidae.

**Mactridae**: The most species-rich family in Mactroidea, dating to the Late Cretaceous (Vokes, 1946; Saul, 1973; Signorelli, 2023), appears to be paraphyletic due to its inclusion of mesodesmatid taxa (Figs. 1 and 2), as noted above.

#### Subfamilies of Mactridae

The subfamilies of Mactridae as presented in Huber (2010); Huber (2015), Carter et al. (2011), Signorelli & Carter (2016), and MolluscaBase (2025) either lack data or are not supported, as detailed below:

**Darininae**: Neither of the two extant species (*Darina rustica* and *D. solenoides*) have been sequenced. Only one extinct genus (*Darcinia*, Eocene) is currently assigned to this subfamily (Signorelli, 2023), leaving a 50-Myr gap in its fossil record.

**Tanysiphoninae**: This monospecific, estuarine subfamily (*Tanysiphon rivalis*) has no known fossil record and has not been sequenced, so its phylogenetic position and status remain unknown.

**Resaniinae**: This monospecific subfamily (*Resania lanceolata*), dating back to the Oligocene (Beu, 2019), is recognized by MolluscaBase (2025), but has been subsumed into Lutrariinae by some authors (*e.g.*, Signorelli, 2019). It has not been sequenced, so its phylogenetic position and status remain unknown.

**Lutrariinae**: This species-rich subfamily is split into two widely separated groups. The first comprises *Lutraria* and *Psammophila* (often considered a subgenus of *Lutraria*), and the other nests within Mactrinae, is paraphyletic with respect to *Mactrinula* and Kymatoxinae, and contains *Meropesta*, *Eastonia* and *Tresus*. The subfamily cannot be sustained without relocating the latter three genera.

**Mactrinae**: The largest subfamily as defined by most recent sources (Bieler, Carter & Coan, 2010; Carter et al., 2011; Signorelli, 2019; and MolluscaBase, 2025) contains genera currently assigned to Mesodesmatidae, Kymatoxinae, and Lutrariinae. More data are needed to frame a phylogenetically robust composition for Mactrinae.

**A new subfamily?** In agreement with the molecular analyses by Liu et al. (2020), Ma et al. (2023) and Ma et al. (2024), the synthesis presented here finds a close relationship between two genera long known to share exclusively internal ligaments, *Rangia* and *Mulinia* (Figs. 1 and 2; ufBSS/sCF = 100/50% and 100/44%, respectively). These genera are in turn closely related to *Spisula* and *Pseudocardium* (Figs. 1 and 2). These findings suggest a new subfamily arrangement for these three taxa (four, if including *Pseudocardium*) and related forms, but additional Atlantic species require sequencing to confirm this hypothesis.

#### Genera of Mactroidea: potential changes in status

The genera of Mactroidea are largely supported by the molecular data, although recent taxonomic decisions that subsume subgenera into more inclusive units receive less support, suggesting that a number of synonymized taxa could be revived as meaningful phylogenetic units. Potential revisions and remaining questions are discussed below.

Mactridae

*Coelomactra*: Our analyses find that *Coelomactra cumingii* and the type species, *C. violacea*, are separate but closely related and firmly nested within *Mactra s.s.* (Figs. 1 and 2). In both the comprehensive and high-coverage trees, one of these species is the closest relative of *Mactra antiquata*, itself having been placed in *Coelomactra* by many authors (Higo, Callomon & Goto, 1999; Okutani, 2000; Ni et al., 2012; Yuan, Kong & Li, 2013; Shen et al., 2016; Yi et al., 2019). Further sequencing of *Coelomactra*, coupled with photographic documentation of voucher specimens, will be essential for resolving its taxonomy.

*Darina:* This genus currently groups *Darina solenoides* (King, 1832) and *D. rustica* (Huber, 2010). Huber’s species was distinguished on the basis of shell shape, particularly the morphology of the anterior and posterior ends. *Darina* has been placed within Mactridae (Huber, 2015; MolluscaBase, 2025), but internal anatomical features, such as separate siphons, suggest closer affinities with Mesodesmatidae. New specimens of *D. solenoides* from Argentina were recently sequenced, with preliminary results showing a clear relation to the genus *Mesodesma*. Accordingly, the validity of the subfamily Darininae is under revision (J. Signorelli, May 2026, unpublished data).

*Hemimactra*: Our analyses suggest that this subgenus of *Spisula* is not an independent lineage, separate from its congeners (Figs. 1 and 2; ufBSS/sCF = 90/28% and 94/30%, respectively); the status of *Hemimactra* should be clarified with more sequence data.

*Mactrotoma*: The synonymy of *Mactrotoma* and *Standella*, both never sequenced, has been widely discussed (Huber, 2010; Huber, 2015; Petit, 2012; Signorelli, 2019; Signorelli, 2021; Valentich-Scott, Coan & Zelaya, 2020). On one hand, Petit (2012) suggested that both genera are based on the same type species and therefore must be considered synonyms. This conclusion was followed by Signorelli (2019), Signorelli (2021). On the other hand, Dall (1903) and later Lamy (1917–1918) considered the type species of *Standella* to be *Mactra fragilis* of Gray, 1853 (= *Mactra pellucida* Gmelin, 1791, and therefore distinct from *Mactra fragilis* Gmelin, 1791). This point of view was followed by Huber (2010), Huber (2015). After reexamination of all related literature (see Bieler & Mikkelsen, 2025 for a useful summary), Huber’s conclusion is adopted here. The genus *Mactrotoma* is kept for Atlantic species, whereas *Standella* should be used for the Indo-Pacific species.

*Mactroderma* and *Micromactra*: *Mactroderma* is considered a subgenus, and *Micromactra* a junior synonym, of *Mactrotoma* by Huber (2010); Huber (2015), and both are treated as synonyms of *Mactrotoma* by other authors (as in MolluscaBase, 2025). *Mactroderma* and *Micromactra* are both considered distinct subgenera of *Standella*, now *Mactrotoma*, in Signorelli (2019). Sequencing of *Mactroderma*, *Mactrotoma* and *Micromactra* species is needed to resolve their status as potentially distinct taxa.

*Oxyperas*: Due to the lack of sequence data for *Oxyperas*, its relationship to other mactrids remains an open question (Signorelli, 2023), as does its incorporation of junior synonyms *Longimactra* and *Pseudoxyperas* (*e.g.*, Beu, 2006; Beu, 2019; Huber, 2015). The potential revival of *Longimactra* as a separate taxon requires special attention; based on morphological evidence, Signorelli (2023) suggests a close relationship among *Oxyperas aspersum* (Sowerby I, 1825), *O. egenum* (Reeve, 1854), and the type species of *Longimactra*, *O. elongatum* (Quoy & Gaimard, 1835), and another among *O. lentiginosum* (Gould, 1852) (= *Mactra triangularis* Lamarck, 1818, type of *Oxyperas*), *O. bernardi* (Pilsbry, 1904), *O. coppingeri* (Smith, 1884), and the type species of *Pseudoxyperas*, *O. proaspersum* (Sacco, 1901). In contrast, Amano, Hamuro & Hamuro (2026) recently cited traits that they regard as distinguishing *Pseudoxyperas* as a valid taxon.

*Psammophila* and *Lutraria*: Two sets of species consistently form sister groups in the high-coverage and comprehensive trees, one representing *Lutraria* (including the type species, *L. lutraria*), and the other representing *Psammophila* (for which the type species *Lutraria* (*Psammophila*) *oblonga* (Gmelin, 1791) has not been sequenced). However, *Lutraria* (*Psammophila*) *sieboldii* Reeve, 1854 appears in the high-coverage tree as sister to the combined *Psammophila* + *Lutraria* clade (Fig. 2), and in the comprehensive tree embedded within *Lutraria* (Fig. 1, with this placement receiving substantial site concordance despite weak bootstrap support; the summarized consensus tree for this analysis suggests the reciprocal monophyly of *Lutraria* and *Psammophila* including *P. sieboldii*). Additional molecular data for *L.* (*P.*) *sieboldii* are needed to resolve this conflict. We also note that both analyses infer a sister relationship between *L.* (*P.*) *australis* and *L.* (*P.*) *rhynchaena*, taxa that were synonymized by Beu (2006: 242) but considered distinct by Huber (2010: 759–760). Again, more molecular data are needed, combined with formal species delimitation analyses, but the two taxa are clearly closely related.

*Pseudocardium*: Potentially represented in our trees by *Mactra sachalinensis* von Schrenck, 1862 *Pseudocardium* was considered an extinct genus by Huber (2015) and MolluscaBase (2025), but as an extant genus or subgenus of *Spisula* by Beu (2004) and Lutaenko & Noseworthy (2012). *M. sachalinensis* von Schrenck, 1862 has been assigned to *Spisula* (*e.g.*, Huber, 2010; Huber, 2015; Lutaenko & Héros, 2011; Ma et al., 2023; MolluscaBase, 2025), and is inferred as sister group to the other members of *Spisula* in the high-coverage tree (Fig. 2; ufBSS/sCF = 86/33%). The comprehensive analysis inferred *Spisula* as more closely related to *Rangia* and *Mulinia* (Fig. 1; ufBSS/sCF = 84/40%) than either is to *M. sachalinensis*. The species was placed in *Pseudocardium* by Woodring, Stewart & Richards (1940: 94) and retained there by many later authors, with *Pseudocardium* a full genus or a subgenus of *Spisula* (Scarlato, 1981; Kafanov, 1991; Okutani, 2000; Kafanov, Ogasawara & Marincovich Jr, 2002; Kafanov & Ogasawara, 2004; Amano, 2024). However, the *P. sachalinensis* specimens illustrated by Okutani (2000) and Lutaenko & Noseworthy (2012) are elongate-ovate, whereas the extinct type species is trigonal with height exceeding length, although this is less extreme in the material figured by Moore (2003). This morphological discrepancy highlights the need for further comparisons between fossil specimens of true *Pseudocardium* and extant *P. sachalinensis*. *Pseudocardium* may well prove to be extinct, as Huber (2015) suggested.

*Raeta*: Ma et al. (2023) sequenced two phylogenetically distinct taxa, *Raeta* (*Raetella*) *pulchella* and *Raeta* sp. The sequences for *Raeta* sp. are from a single individual and probably represent an undescribed species, as the illustrated specimen (Figure 2 of Ma et al., 2023) does not much resemble *Raeta* (*Raetina*) *pellicula* (Reeve, 1854), the only other member of *Raeta sensu lato* mentioned in Chinese waters by Huber (2015). Further, the type species of this genus, *Raeta plicatella* (Lamarck, 1818), is in the western Atlantic far from the source of *Raeta* sp. and has never been sequenced. Thus, not only might *Raeta* sp. of Ma et al. (2023) represent a new species, but further sequencing is needed to confirm whether the Indo-Pacific taxa currently assigned to *Raeta* belong in this genus, or whether they instead require placement in a new one. The status of *Raeta* and its familial position therefore remains uncertain.

*Spisulona*: Beu (2004:166), Beu (2019) and MolluscaBase (2025) considered *Spisulona* a junior synonym of *Spisula s.s.*, but *Spisula discors* and ?*Pseudocardium murchisoni*, two New Zealand species assigned to *Spisulona* by Huber (2010: 758), Huber (2015), Marshall & Spencer (2013: 46) and Signorelli (2023), form a well-supported clade phylogenetically and biogeographically distant from the northeast Atlantic type species of *Spisula s.s.*, *S. solida* (Linnaeus, 1758), and two other species widely regarded as *Spisula s.s.* (Figs. 1 and 2; ufBSS/sCF = 99/68% and 98/68%, respectively). Thus, molecular data currently support retention of *Spisulona* as a valid phylogenetic unit within Mactridae.

Mesodesmatidae

*Taria*: The type species of *Paphies, P. australis* (Gmelin, 1791), is separated from the remaining *Paphies* species and is sister to a mesodesmatid subclade comprising *Atactodea striata* and *Donacilla cornea* (Figs. 1 and 2; ufBSS/sCF = 92/51% and 97/46%, respectively). Unfortunately, no museum voucher is associated with this sequenced specimen, making it impossible to confirm its identity. If additional sequencing, paired with photographic documentation of voucher specimens, confirms this placement of *P. australis*, then *Taria* Gray, 1853 would need to be raised from synonymy. Its type species, *T. stokesii* Gray, 1853, was not fully described or figured, although Beu & De Rooij-Schuiling (1982) designated a neotype (BM(NH) 1852.3.16.292). They consider *T. stokesii* Gray, 1853 to be a junior synonym of *Mya donacina* Spengler, 1793, as do Powell (1979), Huber (2010), Huber (2015), and MolluscaBase (2025). We find that *T. donacina* forms a well-supported clade with two other species assigned to *Paphies* in MolluscaBase (2025) but also assigned to *Taria* by Huber (2010: 762), Huber (2015), (Figs. 1 and 2; ufBSS/sCF = 99/40% and 99/39%, respectively). Thus, molecular data currently support retention of the taxon termed *Taria* by Huber (2010), Huber (2015) and *Paphies* (*Taria*) by MolluscaBase (2025) as a phylogenetically meaningful unit within Mesodesmatidae. Confirmation of the molecular distinctiveness of this small clade, including well-figured voucher specimens, would be desirable. The known fossil record of *Taria* extends to the Late Pliocene (Beu, 2019; Fig. 3). The Lower Miocene *Paphies otaiana* (Eagle, 1999), said by its author to resemble the type species of *Taria*, would create a conflict in the time calibration. However, Eagle’s holotype does not preserve the hinge or other internal structures, and the shell outline is damaged; it is more oblong than triangular (like *Paphies australis*), but also inequilateral (like *Taria donacina*), and thus its affinities are unclear.

**Figure 3:**
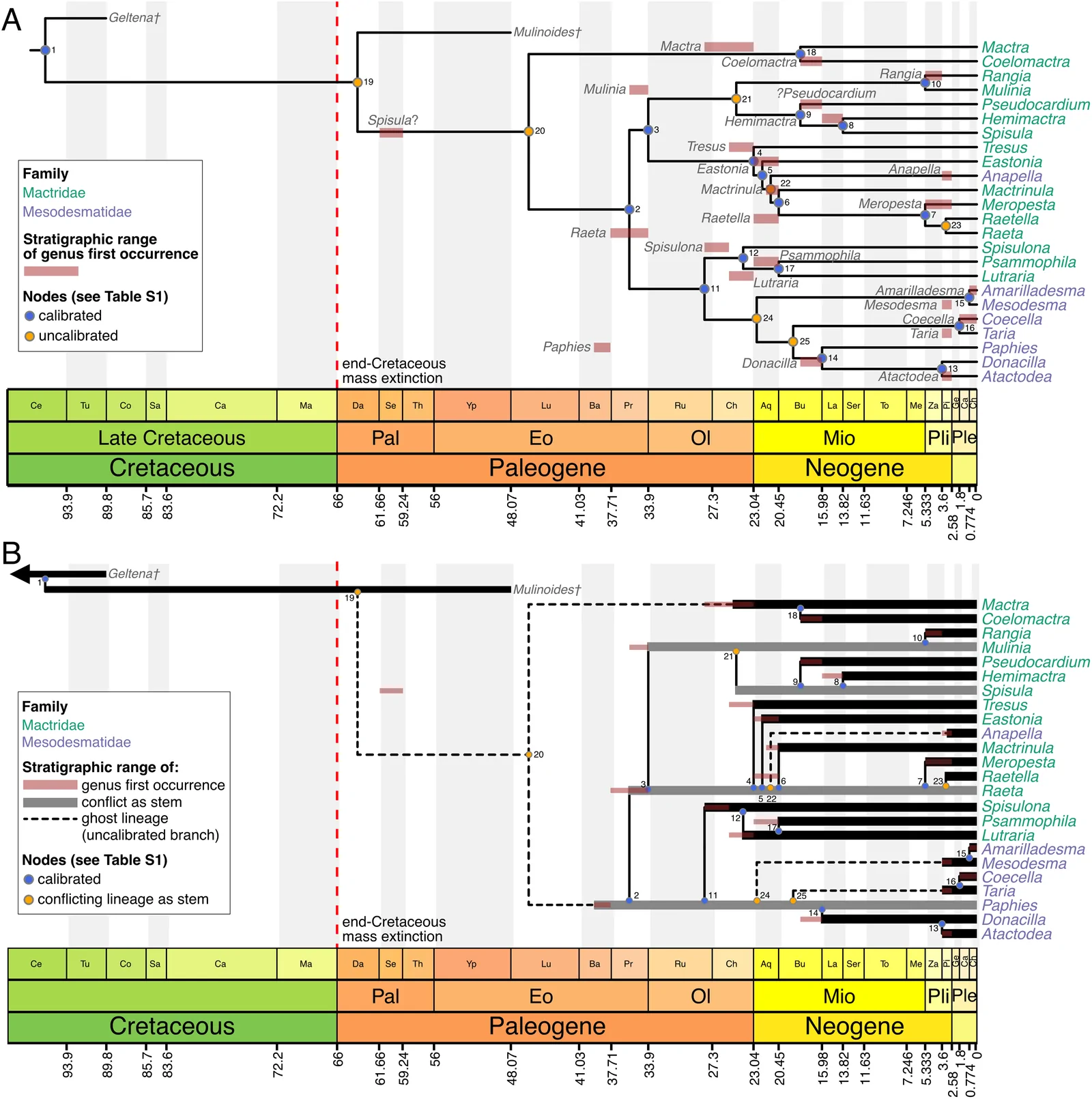
Time-calibrated phylogeny of operational genera based on the topology and taxonomy presented in the high-coverage, three-gene tree (after dropping two tips representing rogue taxa). Blue circles indicate calibrated nodes, with values corresponding to the “Calibration numbers” in Table S1. Gold circles indicate nodes left uncalibrated due to conflicts between the fossil record and the tree topology. Tips are colored by taxonomic family, with Kymatoxinae treated as a subfamily of Mactridae rather than Anatinellidae. (A) Bifurcating branching model; (B) Budding model consistent with the phylogeny in 3A, following the approach of Crouch et al. (2021).

*Soleilletia*: We do not address this monospecific taxon, which is treated as an extant genus of Mesodesmatidae in MolluscaBase (2025). Described from eastern Ethiopia as a Holocene subfossil corbiculid, Mandahl-Barth (1988:112) considers the type to be “a subfossil species of *Caecella* [sic - *Coecella*]”. In contrast, Van Damme & Gautier (1994), who reported additional subfossil material from the neighboring Djibouti Republic, viewed the source deposits as a marine incursion, interpreting the genus and species as an extant marine taxon that “appears to be quite similar to *Ervilia scaliola* Issel 1869”, a species that they considered to be a mesodesmatid, but that is now placed in Tellinoidea: Semelidae. More Djibouti material was reported by Gasse & Fontes (1989), dated at 5,600–5,300 Yr BP, but they inferred a greatly reduced marine influence in the region at that time.

## Phylogenetic estimation of genus divergence times

The inferred molecular topology is broadly consistent with the fossil record (Figs. 2 and 3, Table S1 for fossil details). Limited molecular data from some of the earliest-diverging mactroid lineages still hinder precise estimation of the age of crown Mactroidea (Fig. 3), but we are aware of only one mactroid genus that survives the end-Cretaceous mass extinction, the now-extinct *Mulinoides* (see node 1 in Table S1).

The oldest living mactroid genera appear to be *Mactromeris* (Late Paleocene?), *Oxyperas* (Early Eocene?), *Crassula* (Middle Eocene?), and *Spisula* (Late Paleocene?) (see Tables S1, S2). The fossil records for *Mactromeris* and *Spisula* require particular scrutiny to confirm their earliest occurrences. Further investigation is also required for the following conflicts (Fig. 3), wherein fossils of a putative daughter lineage predate their inferred ancestor, complicating the dating of several nodes (including crown Mactroidea).

(1) *Spisula* and *Mulinia:* These two conflicts between the stratigraphic and molecular data are particularly large and suggest competing hypotheses in which only one of these genera is among the earliest-diverging mactrids (Fig. 3A). The shell outline and hinge plate strongly support *M. mystrosura* (Stilwell & Zinsmeister, 1992) as an early member of *Mulinia*, or at the very least more closely related to *Mulinia* than to extinct stem lineages, likely placing the crown group in the Eocene, albeit of uncertain age within that time interval. Still, none of the current candidates for the oldest fossil *Spisula* or *Mulinia* agree with the molecular phylogeny under a bifurcating model (see Table S1 for information on fossil occurrences). A budding paradigm can accommodate just one of these conflicts, with either *Spisula* or *Mulinia* serving as the ancestral lineage to *Raeta*. In Fig. 3B, we use *Mulinia* to date the split, but consider this matter unresolved. *Mulinia* cannot be used to constrain the stem or crown age of Mactroidea because the oldest known *Mactra s.s.* is younger than the more tipwards split between *Mulinia* and *Paphies*.

(2) Kymatoxinae (*Raeta* and *Raetella*): As with *Spisula* and *Mulinia*, the earliest records for *Raeta* and its subgenus *Raetella* precede those of some ancestral lineages, *i.e., Anapella*, and *Meropesta*. The conflict for *Raeta s.s.* derives from the incompletely known *Labiosa* (*Raeta*) sp. of Clark & Durham, 1946, from the Late Eocene of Colombia. The material is not formally described or figured and so is difficult to evaluate. This gap would be even greater if the Late Cretaceous *Raeta saharica* Collignon, 1971 and *Raeta quadrata* Collignon in Amard, Collignon & Roman, 1981, are validly placed, but both are known only from external impressions lacking preserved hinges, which are necessary for definitive taxonomic assignments. A younger alternative is *Raeta* cf. *R. gardnerae* (Spieker, 1922), from the Late Oligocene Bohio Formation and Caimito Formation, Panama (Woodring, 1982), but again the hinge is not recorded. As Woodring (1982) notes, better-documented records of *Raeta* begin in the Miocene, suggesting that these occurrences may provide more secure time calibrations. The age of *Raetella* sp. from the Early Miocene is similarly problematic (see Table S1, node 23). A budding cladogenesis model can remove one of these conflicts, but the crown age of Kymatoxinae would remain unconstrained.

(3) Crown Mesodesmatidae (*Paphies*): The mid-Eocene age of *Paphies s.s.* (Table S2) is far older than the present geologic ages of most mesodesmatid genera and among the oldest for all of Mactroidea. This age estimate, together with the nested position of *Paphies* within Mesodesmatidae (Figs. 1–3), inhibits calibration of the crown age of the family, as well as the most recent common ancestor of *Paphies* and *Taria*.

## Present-day spatial distribution of sequenced and unsequenced mactroids

Species- and genus-richness of Mactroidea largely mirror the global diversity of Bivalvia as a whole, with a strong global maximum in the tropical West Pacific and pronounced latitudinal gradients, showing decreasing diversity from the tropics towards high latitudes (Figs. 4A, 4D). While the distribution of sequenced species and genera align with this pattern (Figs. 4B, 4E), a closer look at unsequenced species and genera (Figs. 4C, 4F) reveals sampling deficits not just in the tropical West Pacific diversity peak, but also the tropical East Pacific, and (for genera and subgenera) part of the tropical West Atlantic and southern Caribbean, the tropical East Atlantic, southern Australia and New Zealand (see Table 1).

**Figure 4:**
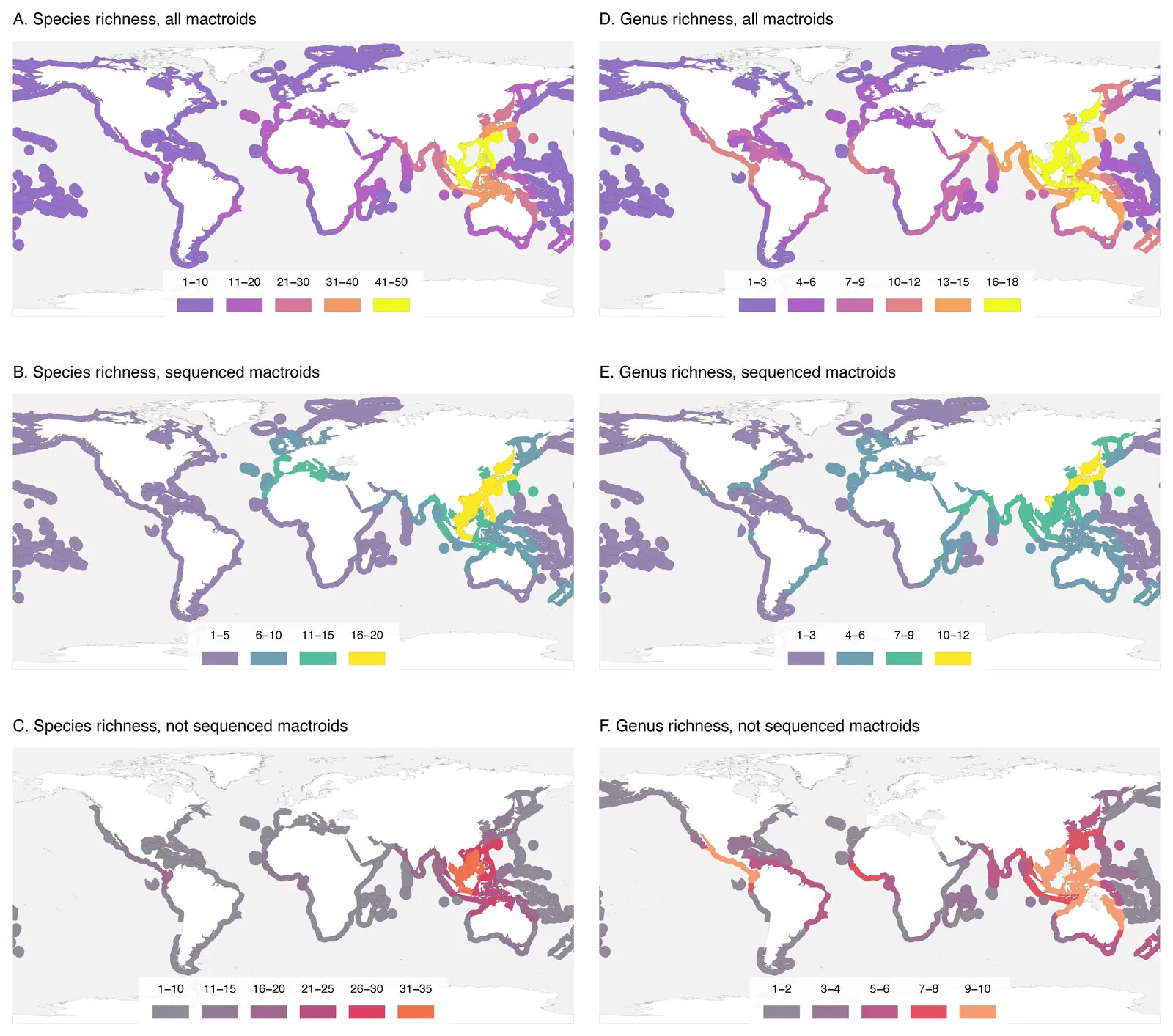
Biogeographic distribution of Mactroidea at the level of species (A–C) and operational genus (D-F), plotted on the shelf ecoregion scheme of Spalding et al. (2007). (A) Species richness; (B) Sequenced species; (C) Unsequenced species; (D) Genus richness; (E) Sequenced genera; (F) Unsequenced genera.

## Discussion

Our synthesis of mactroid Sanger sequencing data suggests a number of potentially polyphyletic genera or subgenera in current taxonomic practice, while supporting others (Figs. 1 and 2). Of the 10 genera and subgenera (here treated as equivalent ranks) having multiple sequenced species, *Mulinia*, *Tresus*, *Coecella*, and *Paphies* constitute monophyletic groups. When subgenera are subsumed within their parent genera, the number of monophyletic groups rises to a total of seven. As summarized in Table 1, at least 64 currently recognized genera and subgenera still lack sequence data. Overall, the available data are encouraging, with a generally high concordance between morphologically and genetically defined units at the genus/subgenus level. However, the taxonomic gaps are not randomly distributed geographically, or in proportion to species richness, with concentrations in the East Pacific and New Zealand (Fig. 4).

In contrast to the genera they comprise, the morphologically defined subfamilies either lack data or are not supported by the molecular data we have. Evidently, homoplasy continues to obscure relationships at higher taxonomic levels in Mactroidea. Existing sequence data are also insufficient to resolve family-level relationships within the superfamily. Transcriptomic data are available for only two species of Mactroidea, confirming the close relationship between Mesodesmatidae and Mactridae, but not resolving relationships beyond that (Lemer, Bieler & Giribet, 2019). The larger clade classified as Mesodesmatidae is nested within Mactridae, as is Kymatoxinae (Figs. 1 and 2), which evidently should be returned to Mactridae from Anatinellidae, as in Bieler, Carter & Coan (2010) and Carter et al. (2011). Among higher taxa within Mactroidea, Cardiliidae, Darininae, Resaniinae, and Tanysiphoninae have yet to be sequenced; molecular data would be valuable in confirming or modifying their systematics. Statistical support declines toward the root in both of our trees (Figs. 1 and 2), indicating that a densely sampled phylogenomic approach may be valuable in this group (as in, *e.g.*, González-Delgado, Rodríguez-Flores & Giribet, 2024; Batistão et al., 2025; Li et al., 2025).

The short internodes near the base of the mactroid trees (Figs. 1 and 2) may indicate that the lineages leading to present-day mactroid diversity originated rapidly during the early Cenozoic, perhaps derived from the single genus known to have survived the 60% loss suffered by bivalves in the end-Cretaceous mass extinction (Edie, Collins & Jablonski, 2025). This interpretation remains tentative, given the limited phylogenetic signal in the present datasets and the number of extant taxa that have yet to be sequenced. Whether these early-diverging branch lengths reflect genuine biological signal should be tested in total-evidence frameworks incorporating extinct Mesozoic and Cenozoic genera. Saul (1973) argued that at least two mactroid lineages survived the extinction to give rise to different segments of the group’s present-day diversity, but our phylogeny does not clearly recover her groupings. Further work is needed to clarify the origin of present-day mactroids relative to the rich pool of extinct taxa and key external drivers of diversity such as the end-Cretaceous extinction and Cenozoic climate changes (Edie et al., 2018).

The distribution of mactroid species and genus richness (Fig. 4) is similar to the biogeography of marine bivalves as a whole; this is not surprising, as mactroids occupy perhaps the most common functional groups within the class, namely infaunal suspension- and deposit-feeding as adults, and planktotrophy as larvae (*e.g.*, Barnes & Morton, 1997; Morton, 2010; Morton, 2016; Huber, 2010: 277). The similarity to the global pattern even extends to one of the most intriguing anomalies in marine biogeography: the presence of more species along the relatively linear, temperate coasts of southern Japan and southeast Australia than in the more two-dimensional, tropical Caribbean region. This contrast may reflect a combination of the Late Cenozoic extinction pulse in the Caribbean and spillover from the tropical West Pacific hotspot (Jablonski et al., 2017). In any case, the pattern disrupts any simple global relationship between regional diversification and sea-surface temperature and merits further investigation. The global distribution of unsequenced genera in particular highlights regions that are important for further sampling and accompanying documentation (in order of declining numbers of unsequenced taxa): the tropical East Pacific and the tropical West Pacific with some adjoining regions (including New South Wales, northwest Australia, and the eastern Indian Ocean).

An attempt to time calibrate the mactroid phylogeny shows a number of conflicts between taxon ages in the fossil record and the molecular tree topology, even when a budding calibration is used instead of a strictly bifurcating one (Fig. 3; see Marshall, 2019; Crouch et al., 2021; Parins-Fukuchi & Saulsbury, 2025 for discussion). Some of these conflicts likely result from difficulties in taxonomic placement for poorly preserved fossil material, but others may arise from the large number of extant and extinct genera still missing from the phylogeny (as in, *e.g.*, the long ghost lineage of *Mactra s.s.*). As currently known, 11 mactroid genera are apparently confined to the Mesozoic, approximately 26 confined to the pre-Holocene Cenozoic (see Signorelli, 2023 and Tables S1 and S2), and another 40 extant genera and subgenera are absent from the phylogeny. The systematic questions raised by our findings underscore the urgent need for molecular data to be supported by voucher specimens, ensuring that resulting taxonomic conclusions are robust and verifiable. These sampling gaps and uncertainties inevitably produce conflicts, especially when unevenly distributed across biogeographic regions (Fig. 4F). However, by pinpointing such conflicts we hope to guide future research into the dynamics of this economically and ecologically important clade.

## Conclusion

This integrative inventory of molecular phylogenetic coverage and implications for significant subclades within higher taxa can be used to guide current practice, as well as taxonomic and biogeographic sequencing priorities. Though available molecular data for Mactroidea have clarified many internal relationships within the superfamily, a number of open questions remain (see Table 1). Similar analyses of other groups, pinpointing the most consequential targets for sequencing within and beyond Bivalvia, would be valuable going forward.

## Supplemental Information

10.7717/peerj.21681/supp-1 Supplemental Information 1 Supplemental Tables Table S1 (paleontological evidence for node calibrations), Table S1 (potential oldest extant mactroids, not included in Table S1), and associated references.

## Additional Information and Declarations

### Data Availability

The following information was supplied regarding data availability: The raw data and code are available at Zenodo: - Ghezelayagh, A., Edie, S., & Jablonski, D. (2026). Data and code from: Synthesis of molecular data on Mactroidea (Bivalvia): phylogenetic implications and open questions [Data set]. In PeerJ. Zenodo. https://doi.org/10.5281/zenodo.21728549

## References

[ref-1] Altschul SF, Gish W, Miller W, Myers EW, Lipman DJ (1990). Basic local alignment search tool. Journal of Molecular Biology.

[ref-2] Amano K (2024). Speciation, extinction and survival of molluscan species in the marginal sea–Plio-Pleistocene Omma-Manganji fauna in the Japan Sea borderland. Bulletin of the Mizunami Fossil Museum.

[ref-3] Amano K, Hamuro T, Hamuro M (2026). Crassatellid and mactrid Bivalves from the Lower Miocene Kurosedani Formation in Toyama City, Toyama Prefecture, central Japan. Bulletin of the National Museum of Nature and Science Series D (Paleontology and Anthropology).

[ref-4] Amard B, Collignon M, Roman J (1981). Étude stratigraphique et paléontologique du Crétacé supérieur et Paléocène du Tinrhert-W et Tademait-E (Sahara algérien). Documents des Laboratoires de Géologie de la Faculté des Sciences de Lyon, Hors Série.

[ref-5] Barnes PAG, Morton B (1997). The functional morphology of *Mactrinula reevesii* (Bivalvia: Mactroidea) in Hong Kong: Adaptations for a deposit-feeding lifestyle. Journal of Zoology.

[ref-6] Batistão A, Audino J, Frigyik E, Tallarico L, Introíni G, Passos F, Giribet G (2025). Ultraconserved element-based phylogenomics and siphonal traits illuminate the evolution of tellinoidean clams (Mollusca: Bivalvia: Tellinoidea). Zoological Journal of the Linnean Society.

[ref-7] Bennett DJ, Hettling H, Silvestro D, Zizka A, Bacon CD, Faurby S, Vos RA, Antonelli A (2018). Phylotar: an automated pipeline for retrieving orthologous DNA sequences from GenBank in R. Life.

[ref-8] Beu AG (2004). Marine Mollusca of oxygen isotope stages of the last 2 million years in New Zealand. Part 1: Revised generic positions and recognition of warm-water and cool-water migrants. Journal of the Royal Society of New Zealand.

[ref-9] Beu AG (2006). Marine Mollusca of oxygen isotope stages of the last 2 million years in New Zealand. Part 2. Biostratigraphically useful and new Pliocene to Recent bivalves. Journal of the Royal Society of New Zealand.

[ref-10] Beu AG (2019). Catalogue of New Zealand Cenozoic marine Mollusca. Pers. comm. to DJ, 2019; posthumous manuscript now being completed and prepared for publication by Henry Gard, GNS Science, Lower Hutt, New Zealand.

[ref-11] Beu AG, De Rooij-Schuiling LA (1982). Subgeneric classification of New Zealand and Australian species of Paphies Lesson (Bivalvia: Mesodesmatidae), and names for the two species of tuatua in New Zealand. New Zealand Journal of Zoology.

[ref-12] Bieler R, Carter J, Coan EV (2010). Classification of bivalve families. Pp. 113-133, in: P. Bouchet & J.-P. Rocroi, 2010. Nomenclator of bivalve families. Malacologia.

[ref-13] Bieler R, Mikkelsen PM (2025). A taxonomic update for Seashells of Southern Florida–Bivalves (Mikkelsen and Bieler, 2007) with nomenclatural notes on other bivalve species. Nautilus.

[ref-14] Capella-Gutiérrez S, Silla-Martínez JM, Gabaldón T (2009). trimAl: a tool for automated alignment trimming in large-scale phylogenetic analyses. Bioinformatics.

[ref-15] Carter JG, Altaba CR, Anderson LC, Araujo R, Biakov AS, Bogan AE, Campbell DC, Campbell M, Chen J, Cope JCW, Delvene G, Dijkstra HH, Fang Z, Gardner RN, Gavrilova VA, Goncharova IA, Harries PJ, Hartman JH, Hautmann M, Hoeh WR, Hylleberg J, Jiang B, Johnston P, Kirkendale L, Kleemann K, Koppka J, Kříž J, Machado D, Malchus N, Márquez-Aliaga A, Masse J-P, McRoberts CA, Middelfart PU, Mitchell S, Nevesskaja LA, Özer S, Pojeta Jr J, Polubotko IV, Pons JM, Popov S, Sánchez T, Sartori AF, Scott RW, Sey II, Signorelli JH, Silantiev VV, Skelton PW, Steuber T, Waterhouse JB, Wingard GL, Yancey T (2011). A synoptical classification of the Bivalvia (Mollusca). University of Kansas, Paleontological Institute, Paleontological Contributions.

[ref-16] Crouch NMA, Edie SM, Collins KS, Bieler R, Jablonski D (2021). Calibrating phylogenies assuming bifurcation or budding alters inferred macroevolutionary dynamics in a densely sampled phylogeny of bivalve families. Proceedings of the Royal Society B.

[ref-17] Dall WH (1903). Contributions to the Tertiary fauna of Florida, with especial reference to the Miocene silex–beds of Tampa and the Pliocene beds of the Caloosahatchie River.

[ref-18] Eagle MK (1999). Six new fossil bivalves from the Early Miocene of Auckland and Northland, New Zealand. Records of the Auckland Museum.

[ref-19] Edie SM, Collins KS, Jablonski D (2025). The end-Cretaceous mass extinction restructured functional diversity but failed to configure the modern marine biota. Science Advances.

[ref-20] Edie SM, Huang S, Collins KS, Roy K, Jablonski D (2018). Loss of biodiversity dimensions through shifting climates and ancient mass extinctions. Integrative and Comparative Biology.

[ref-21] Gasse F, Fontes JC (1989). Palaeoenvironments and palaeohydrology of a tropical closed lake (Lake Asal, Djibouti) since 10,000yr BP. Palaeogeography, Palaeoclimatology, Palaeoecology.

[ref-22] González-Delgado S, Rodríguez-Flores PC, Giribet G (2024). Testing ultraconserved elements (UCEs) for phylogenetic inference across bivalves (Mollusca: Bivalvia). Molecular Phylogenetics and Evolution.

[ref-23] Guindon S, Dufayard JF, Lefort V, Anisimova M, Hordijk W, Gascuel O (2010). New algorithms and methods to estimate maximum-likelihood phylogenies: assessing the performance of PhyML 3.0. Systematic Biology.

[ref-24] Higo S, Callomon P, Goto Y (1999). Catalogue and bibliography of the marine shell-bearing Mollusca of Japan.

[ref-25] Hoang DT, Chernomor O, von Haeseler A, Minh BQ, Vinh LS (2018). UFBoot2: improving the ultrafast bootstrap approximation. Molecular Biology and Evolution.

[ref-26] Huang S, Edie SM, Collins KS, Crouch NMA, Roy K, Jablonski D (2023). Diversity, distribution and intrinsic extinction vulnerability of exploited marine bivalves.. Nature Communications.

[ref-27] Huber M (2010). Compendium of bivalves.

[ref-28] Huber M (2015). Compendium of bivalves 2. A full-color guide to the remaining seven families.

[ref-29] Jablonski D, Huang S, Roy K, Valentine JW (2017). Shaping the latitudinal diversity gradient: new perspectives from a synthesis of paleobiology and biogeography. American Naturalist.

[ref-30] Kafanov AI (1991). Bivalve molluscs and faunistic biogeography of the Northern Pacific.

[ref-31] Kafanov AI, Ogasawara K (2004). Neogene and Paleogene Molluscan (Bivalvia) Cenozones of the Russian Northeast. Science Reports of the Institute of Geoscience, University of Tsukuba B.

[ref-32] Kafanov AI, Ogasawara K, Marincovich Jr L (2002). Checklist of the Cenozoic marine Bivalvia (Mollusca) of Northeastern Asia (Russian Far East), 1893–1999: a taxonomical summary. Science Reports of the Institute of Geoscience, University of Tsukuba B.

[ref-33] Kalyaanamoorthy S, Minh BQ, Wong TKF, von Haeseler A, Jermiin LS (2017). ModelFinder: fast model selection for accurate phylogenetic estimates. Nature Methods.

[ref-34] Katoh K, Standley DM (2013). MAFFT multiple sequence alignment software version 7: improvements in performance and usability. Molecular Biology and Evolution.

[ref-35] Lamy E (1917–1918). Révision des Mactridae vivants du Museum d’ Histoire Naturelle de Paris. Journal de Conchyliologie.

[ref-36] Lemer S, Bieler R, Giribet G (2019). Resolving the relationships of clams and cockles: dense transcriptome sampling drastically improves the bivalve tree of life. Proceedings of the Royal Society B.

[ref-37] Li YX, Ip JCH, Chen C, Xu T, Zhang Q, Sun Y, Ma PZ, Qiu JW (2025). Phylogenomics of Bivalvia using ultraconserved elements reveal new topologies for Pteriomorphia and Imparidentia. Systematic Biology.

[ref-38] Liu J, Zeng Q, Wang H, Teng M, Guo X, Bao Z, Wang S (2020). The complete mitochondrial genome and phylogenetic analysis of the dwarf surf clam *Mulinia lateralis*. Mitochondrial DNA Part B.

[ref-39] Liza CR, Stotz WB, Haye PA (2024). Mito-nuclear discordance and phylogeography of the surf clam *Mesodesma donacium* along the Southeast Pacific coast. Zoologica Scripta.

[ref-40] Lutaenko KA, Héros V (2011). Type material of bivalve mollusks collected during *Vénus* Voyage (1836–1839) from Kamchatka, Russia, in the Muséum National d’Histoire Naturelle, Paris. Malacologia.

[ref-41] Lutaenko KA, Noseworthy RG (2012). Catalogue of the living Bivalvia of the continental coast of the Sea of Japan (East Sea), (p. 247).

[ref-42] Ma P, Liu Y, Wang J, Chen Y, Zhang Z, Zhang T, Wang H (2023). Comparative analysis of the mitochondrial genomes of the family Mactridae (Mollusca: Venerida) and their phylogenetic implications. International Journal of Biological Macromolecules.

[ref-43] Ma P, Liu Z, Li Z, Sun X, Zhou L, Wu X, Wu B (2024). Sequencing of the complete mitochondrial genome of the big brown Mactra Clam, Mactra grandis (Venerida: Mactridae). Animals.

[ref-44] Mandahl-Barth G (1988). Studies on African freshwater bivalves.

[ref-45] Marshall BA, Spencer HG (2013). Comments on some taxonomic changes affecting marine Bivalvia of the New Zealand region recently introduced in Huber’s *Compendium of bivalves*, with some additional taxonomic changes. Molluscan Research.

[ref-46] Marshall CR (2019). Using the fossil record to evaluate timetree timescales. Frontiers in Genetics.

[ref-47] Minh BQ, Schmidt HA, Chernomor O, Schrempf D, Woodhams MD, Von Haeseler A, Lanfear R (2020). IQ-TREE 2: new models and efficient methods for phylogenetic inference in the genomic era. Molecular Biology and Evolution.

[ref-48] Mo YK, Lanfear R, Hahn MW, Minh BQ (2023). Updated site concordance factors minimize effects of homoplasy and taxon sampling. Bioinformatics.

[ref-49] MolluscaBase (2025). MolluscaBase. http://www.molluscabase.org.

[ref-50] Moore EJ (2003). Tertiary marine pelecypods of California and Baja California, Chapter F: crassatellidae, Cardiidae, Mactridae, Mesodesmatidae, Solenidae, Pharidae. Published by the author. https://www.molluscabase.org/aphia.php?p=sourcedetailsid=512619.

[ref-51] Moroz SA (1972). The Paleocene fauna of Mollusca from the Dnieper-Donetsk Basin.

[ref-52] Morton B (2010). Form and functional morphology of *Raetellops pulchella* (Bivalvia: Mactridae): an example of convergent evolution with anomalodesmatans. Invertebrate Biology.

[ref-53] Morton B (2016). The biology and functional morphology of the high-energy beach dwelling *Paphies elongata* (Bivalvia: Mactroidea: Mesodesmatidae). Convergence with the surf clams (*Donax*: Tellinoidea: Donacidae). Journal of Natural History.

[ref-54] Ni L, Li Q, Kong L, Huang S, Li L (2012). DNA barcoding and phylogeny in the family Mactridae (Bivalvia: Heterodonta): evidence for cryptic species. Biochemical Systematics and Ecology.

[ref-55] Okutani T (2000). Marine mollusks in Japan.

[ref-56] Parins-Fukuchi CT, Saulsbury JG (2025). The consequences of budding speciation on trees. Systematic Biology.

[ref-57] Petit RE (2012). John Edward Gray (1800–1875): his malacological publications and molluscan taxa. Zootaxa.

[ref-58] Portik DM, Wiens JJ (2020). SuperCRUNCH: a bioinformatics toolkit for creating and manipulating supermatrices and other large phylogenetic datasets. Methods in Ecology and Evolution.

[ref-59] Powell AWB (1979). New Zealand Mollusca.

[ref-60] R Core Team, 2025 (2025). R: a language and environment for statistical computing. https://www.r-project.org.

[ref-61] Saul LR (1973). Evidence for the origin of the Mactridae (Bivalvia) in the Cretaceous. University of California Publications in Geological Sciences.

[ref-62] Saul LR (1989). California Late Cretaceous donaciform bivalves. Veliger.

[ref-63] Sayers EW (2010). Entrez Direct: E-utilities on the UNIX Command Line. https://www.ncbi.nlm.nih.gov/books/NBK179288/.

[ref-64] Scarlato OA (1981). Bivalve mollusks of temperate latitudes of the western part of the Pacific Ocean.

[ref-65] Shen X, Meng X, Tian M, Yan B, Cheng H, Lu W, Chai Y (2016). The first mitochondrial genome of *Coelomactra antiquata* (Mollusca: Veneroida: Mactridae) from Guangxi (China) and potential molecular markers. Mitochondrial DNA Part A.

[ref-66] Shen X, Meng XP, Chu K, Zhao NN, Tian M, Liang M, Hao J (2014). Comparative mitogenomic analysis reveals cryptic species: a case study in Mactridae (Mollusca: Bivalvia). Comparative Biochemistry and Physiology Part D: Genomics and Proteomics.

[ref-67] Signorelli JH (2019). The Superfamily Mactroidea (Mollusca: Bivalvia) in American waters: an illustrated catalogue of Recent species.

[ref-68] Signorelli JH (2021). The Family Mactridae Lamarck, 1809 (Bivalvia: Cardiida) in South African waters. Malacologia.

[ref-69] Signorelli JH (2023). Catalogue of fossil genera of Mactridae (Mollusca: Bivalvia). Journal of Paleontology.

[ref-70] Signorelli JH, Carter JG (2016). The Anatinellidae and Kymatoxinae: a reassessment of their affinities within the superfamily Mactroidea (Mollusca, Bivalvia). American Malacological Bulletin.

[ref-71] Signorelli JH, Raven JGM (2018). Current knowledge of the family Cardiliidae (Bivalvia, Mactroidea). Journal of Paleontology.

[ref-72] Smith SA, O’Meara BC (2012). treePL: divergence time estimation using penalized likelihood for large phylogenies. Bioinformatics.

[ref-73] Spalding MD, Fox HE, Allen GR, Davidson N, Ferdaña ZA, Finlayson M, Halpern BS, Jorge MA, Lombana A, Lourie SA, Martin KD, McManus E, Molnar J, Recchia CA, Robertson J (2007). Marine ecoregions of the world: a bioregionalization of coastal and shelf areas. BioScience.

[ref-74] Streicher JW, Schulte JA, Wiens JJ (2016). How should genes and taxa be sampled for phylogenomic analyses with missing data? An empirical study in iguanian lizards. Systematic Biology.

[ref-75] Taylor JD, Williams ST, Glover EA, Dyal P (2007). A molecular phylogeny of heterodont bivalves (Mollusca: Bivalvia: Heterodonta): new analyses of 18S and 28S rRNA genes. Zoologica Scripta.

[ref-76] Valentich-Scott PV, Coan EV, Zelaya DG (2020). Bivalve seashells of western South America. Santa Barbara, CA: Santa Barbara Museum of Natural History Monographs.

[ref-77] Van Damme D, Gautier A (1994). A malacological mini-mystery almost solved: the genus *Soleilletia* Bourguignat 1885 (Bivalvia). Journal of Molluscan Studies.

[ref-78] Vokes HE (1946). Contributions to the paleontology of the Lebanon Mountains, Republic of Lebanon. Part 3, The pelecypod fauna of the Olive Locality (Aptian) at Abeih. Bulletin of the American Museum of Natural History.

[ref-79] Wong TKF, Ly-Trong N, Ren H, Banos H, Roger AJ, Susko E, Bielow C, De Maio N, Goldman N, Hahn MW, Huttley G, Lanfear R, Minh BQ (2026). IQ-TREE 3: phylogenomic inference software using complex evolutionary models. Molecular Biology and Evolution.

[ref-80] Woodring WP (1982). Geology and paleontology of Canal Zone and adjoining parts of Panama. Description of Tertiary mollusks (pelecypods: Propeamussiidae to Cuspidariidae; additions to families covered in P306-E; additions to gastropods; cephalopods). U.S. Geological Survey Professional Paper.

[ref-81] Woodring WP, Stewart R, Richards RW (1940). Geology of the Kettleman Hills oil field, California: Stratigraphy, paleontology, and structure. U.S. Geological Survey Professional Paper.

[ref-82] Yi L, Ma KY, Qin J, Chu KH, Shen X, Meng X (2019). Insights into cryptic diversity and adaptive evolution of the clam *Coelomactra antiquata* (Spengler, 1802) from comparative transcriptomics. Marine Biodiversity.

[ref-83] Yuan Y, Kong L, Li Q (2013). Mitogenome evidence for the existence of cryptic species in *Coelomactra antiquata*. Genes & Genomics.

