# Supplementary material for: Synthesis of molecular data on Mactroidea (Bivalvia): phylogenetic implications and open questions
Source: PeerJ. 2026 Sep 30;14:e21681. doi: 10.7717/peerj.21681 (PMC13633688; doi:10.7717/peerj.21681)
Supplement: Supplementary file 1 [file peerj-14-21681-s001.docx]

**Table S1. Paleontological evidence for node calibrations.** Nodes 19-25 are left uncalibrated due to conflicts between the tree topology and fossil ages.

| **Node**  **number** | **Node description** | **Dated taxon** | **Age max (Ma)** | **Age min (Ma)** | **Comments** |
| --- | --- | --- | --- | --- | --- |
| 1 | Stem group precursor of crown group Mactroidea | *Mulinoides* | 96.1 | 93.9 | *M.* cf. *M. olbrechtsi* Dartevelle & Freneix, 1957, Upper Cenomanian or lowermost Turonian part of Cotinguiba Formation, Laranjeiras 28, Brazil (Ayoub-Hannaa et al., 2019, although this record is based on a single, poorly preserved specimen that may be *Cymbophora*). Next oldest is *M. olbrechtsi* Dartevelle & Freneix, 1957, Lower-Middle Santonian Couche 68, Bulu-Zambi, Congo (age from Freneix, 1959:65, 91; potentially also *Cymbophora*). Type species *M. chicama* Olsson, 1944, Upper Campanian-Lower Maastrichtian Tortuga Formation, Paita, Peru (type species, age from Mourier et al., 1988:157). |
| 2 | *Mulinia*, younger than *Paphies* | *Mulinia* | 35.85 | 33.9 | *M. mystrosura* (Stilwell & Zinsmeister, 1992), Lower Lutetian (?) Unit 5 [47.8-44.6 Ma], La Meseta Formation, Seymour Island (age of unit from Dutton et al., 2002, but could be as old as Upper Ypresian (Ivany et al., 2008; Charnelli et al., 2024), as young as mid-Priabonian (Amenabar et al., 2022); “closely similar in shape and in hinge characters to the South American genus *Mulinia*, especially to the relatively large type species, *M. edulis* (King, 1832), with its similarly “rotated” resilifer, and a position in *Mulinia* seems appropriate”- Beu, 2009:216. |
| 3 | *Mulinia*, younger than *Raeta* | *Mulinia* | 35.85 | 33.9 | See node 2. See node 2. |
| 4 | *Tresus*, younger than *Raeta* | *Tresus* | 25.57 | 23.03 | *T. saxadomoides* (Clark, 1918) [spelled *saxidomoides* in plate caption], Upper Oligocene (?) San Ramon Formation, UC loc 1131. Contra Costa County, CA (Moore, 2003, who gives age as "Miocene(?)" (her query); uppermost Oligocene or lowest Miocene in Amano et al. 1993:245; "late Oligocene and (or) early Miocene" in Powell & Rugh 2025; species not listed in Hall 2002, whose oldest occurrence of the genus is a Lower Miocene Tresus sp.). |
| 5 | *Eastonia*, younger than *Raeta* | *Eastonia* | 23.03 | 20.44 | *E. mitis* (Mayer, 1867), Lower Aquitanian stratotype, France (Eames & Clarke, 1967; Alvinerie et al., 1977, Glibert & van de Poel, 1970, who give Aquitanian localities as Leognan (Le Thil) (Bordelais) and Saint-Avit (Landes)); also Aquitanian Pectunculus-Sande, Budapest, Hungary (Schultz, 2003:673), and Lower Burdigalian Sables a Mactres, Martillac (Girdonde), France (Lesport & Cahuzac, 2005). Even if omitting *Raeta*, this node would be dated using *Raetella*, which is the same age as *Eastonia.* |
| 6 | *Mactrinula*, younger than *Raeta* | *Mactrinula* | 21.74 | 20.44 | *M. reevesii* (Gray, 1837) Upper Aquitanian-Lower Burdigalian Pyawbwe Formation, Myanmar (Licht et al., 2014; Ridd & Racey, 2015), and Middle Miocene Boka Bil Stage (Kyaukkok equivalent), Surma Valley, Assam (Eames, 1970). Younger is *M.* cf. *M. plicataria* (Linnaeus, 1758), Middle Miocene Parkini Mudstone, Pasni section, Pakistan (Crame, 1984). Even if omitting *Raeta*, this node would still be dated using *Mactrinula.* |
| 7 | *Meropesta*, younger than *Raeta* | *Meropesta* | 5.33 | 2.59 | *M. nicobarica* (Gmelin, 1791), unnamed Pliocene unit, Mount Wangi and Cipamali, in Bumiayu area, Java (Oostingh, 1931, 1935:196 in *Standella* (*Eastonia*), but species reassigned (Oliver, 1992; Lamprell & Whitehead, 1992; Vongpanich, 2000; Huber, 2010). Even if omitting *Raeta*, this node would still be dated using *Meropesta.* |
| 8 | *Spisula* age uncertain, but assumed to be older than sister *Hemimactra* | *Hemimactra* | 15.97 | 13.82 | *H. densa* (Dall, 1898 [1890-1903]), Langhian Oak Grove Sands of Alum Bluff Group, Florida (Gardner, 1926-1928; has low umbo, more ovate shape and striated laterals of *Hemimactra* rather than *Leptomactra*, cf. Ward, 1992). Also *H. chesapeakensis* (Glenn, 1904), Langhian, Bed 10, Plum Point Memb, Calvert Formation, Plum Point, Calvert Co., Maryland (Petuch & Drolshagen, 2010:63, age from Zimmt et al, 2022). Note: *Spisula* (*Hemimactra*) *dodona* Dall, 1898 and *S.* (*H.*) *valhosierr* Gardner, 1926-1928, Burdigalian Alum Bluff Formation, Florida were put in "Section *Mactromeris*" by Gardner, 1926-1928, but the hinge does not resemble the type species of *Hemimactra* (see Signorelli, 2019). |
| 9 | *Spisula* age uncertain, but assumed to be older than *Pseudocardium* | *Pseudocardium* | 18.21 | 15.97 | *P. densatum densatum* (Conrad, 1857), Burdigalian-Lower Langhian Temblor Formation, Coalinga region, CA (Moore, 2003, citing Adegoke, 1969:119, Moore has Temblor as Oligocene-Lower Miocene, revised age from Valenzuela-Toro et al., 2024 [senior synonym of the type species, *Cardium gabbii* Rémond, 1863, according to Moore, 2003; Signorelli, 2023]). Note: extant species tentatively "*Mactra*" *sachalinensis* von Schrenck, 1882, following Woodring et al., 1940:94; put in *Spisula s.s.* by Huber, 2010, 2015 and Lutaenko & Héros, 2011, but made a junior synonym of *Pseudocardium* *sybillae* (Valenciennes, 1858) by Higo et al., 1999, and kept in *Pseudocardium* by Scarlato, 1981; Kafanov, 1991; Okutani, 2000; Kafanov & Ogasawara, 2004; Amano, 2024, and this species does not form a clade with the type species of *Spisula* in our comprehensive, 8-gene analysis. Genus may be extinct. |
| 10 | *Rangia*, younger than sister *Mulinia* | *Rangia* | 5.33 | 3.6 | *R. clathrodonta* (Conrad, 1833), Lower Pliocene Zone 2, Yorktown Formation, Williamsburg and Yorktown, VA (Campbell, 1993). Note: Thanetian base in Sepkoski, 2002 was based on *Praerangia minuscula* Cossmann, 1908, type species of this valid, extinct genus (see Signorelli, 2023). |
| 11 | *Spisulona*, younger than *Paphies* | *Spisulona* | 28.1 | 25.57 | *S.* n. sp., Duntroonian (Lower & Middle Chattian; Raine et al., 2015), Pomahaka Formation, Pomahaka, New Zealand (Beu, 2019 as *Spisula*). Younger is *S. murchisoni* (Deshayes in Reeve, 1854), Tongaporutuan? [~Tortonian], New Zealand (Beu, 2019 as *Pseudocardium?*, put in *Spisula* (*Spisulona*) by Huber, 2010:758 and Marshall & Spencer, 2013:46, in *Mactra* by Beu & Maxwell, 1990:399 and Beu, 2004:171). Younger is *S.* n. sp., unnamed Opoitian (=Zanclean, Raine et al., 2015) unit, Kaawa Creek, New Zealand (Beu, 2019, as *Spisula* n. sp. but he uses *Spisulona* as a junior synonym). |
| 12 | *Lutraria*, younger than *Spisulona* | *Lutraria* | 25.57 | 23.03 | *L. sanna* Basterot, 1825, Upper Chattian Pielach Formation, Newinden bei Melk, Austria (Harzhauser & Mandic, 2001; Schultz, 2003), see Hölzl, 1962 for Chattian records in Bavaria; also in type Aquitanian, France (Alvinerie et al., 1977); also *L. praeangusta* (Hölzl, 1962), unnamed Chattian unit, Kalvarienberg, Bavaria. |
| 13 | *Atactodea*, younger than sister *Donacilla* | *Atactodea* | 3.6 | 2.59 | *A. striata* (Gmelin, 1791), unnamed Upper Pliocene unit, Niue Island (Paulay, 1996). Also *A. cuneata* (Lamarck, 1818), Upper Pliocene Point Ellen Formation, St Vincent Basin, Australia (Darragh, 2024) and *A. erycinaea* (Lamarck, 1818), Upper Pliocene part of Cameron Inlet Formation, Wingaroo Bore No. 1, Flinders Island, Tasmania (Darragh in Sutherland & Kershaw, 1971, as *Donacilla erycinaea*; Darragh, 2024 confirms Late Pliocene age). |
| 14 | *Donacilla*, younger than *Paphies* | *Donacilla* | 18.21 | 15.97 | *D. cornea* (Poli, 1791), Upper Burdigalian, Karpatium, Niederkreuzstetten, Austria (Schultz, 2003, who also has questionable lower Burdigalian [Lower Ottnangium] record, Alt-Wartenberg bei Vocklabruck, Austria); also unnamed Lower Badenian (=Langhian) unit, Tarnene, Bulgaria (Studencka et al., 1998; see Marasti, 1991 for Italian records). |
| 15 | *Amarilladesma*, younger than sister *Mesodesma* | *Amarilladesma* | 0.78 | 0.01 | *A. mactroides* (Reeve, 1844), Upper Pleistocene (MIS 5e), San Antonio Este, Argentina (Charo et al., 2014). |
| 16 | *Coecella*, younger than sister *Taria* | *Coecella* | 1.81 | 0.01 | *C. chinensis* (Deshayes, 1855), Pleistocene Tungyuping Formation, Penghu Island, Taiwan (Masuda & Huang, 1990a), also Pleistocene Otsu Formation, Otsu [now in Yokosuka City], Kanagawa Prefecture, Japan (Oyama, 1973). |
| 17 | *Psammophila*, younger than sister *Lutraria* | *Psammophila* | 23.03 | 20.44 | *P. magna soror* (Mayer, 1867), Aquitanian, Haute-Baviere, France (Schultz, 2003:671, who has other Aquitanian locs for this species; van Urk, 1980 puts *L. magna* in *Lutraria s.s.*, but on p. 238 & 252 states that "it may be useful to denote it as *L*. (*Psammophila*)" and Beu, 2006:251 puts *L. magna* in *Psammophila*. Also *P. nenokamiensis* (Kanno, 1958), lower Aquitanian Mushan Formation, North Taiwan (Kanno & Chung, 1973; Chung & Kanno, 1975; Masuda & Huang, 1990a) and Lower Miocene Nenokami Formation, Chichibu Basin, Saitama Prefecture, Japan (type locality). Also *P.* sp. A, Early Miocene Tsouho Formation, Hsintie, Taiwan (Masuda & Huang, 1990b); perhaps also *P. trapezoidalis* (Powell & Bartrum, 1929), Otaian (Upper Aquitanian-Lower Burdigalian), Waiheke Island, Auckland, and Altonian (Upper Burdigalian) Castle Hill Basin, Canterbury, New Zealand (Beu, 2006:251). Perhaps also. *P*. aff. *P. australis* (Reeve, 1844), unnamed Lower Miocene unit, Inhacuaia and Mongorro, Mozambique (Cox, 1939, as *Lutraria* cf. *philippinarum* Reeve, 1844). |
| 18 | *Coelomactra*, younger than sister *Mactra* | *Coelomactra* | 18.21 | 15.97 | *C. iwatensis* (Hatai, 1940), uppermost Lower Miocene Shiratori Beds (=Tsate Sandstone Member of Kadonosawa Formation, Iwate Prefecture, Japan (Matsubara 1997; Itoh & Watanabe 2000 date Kadonosawa Formation as Langhian, i.e. lower Middle Miocene). |
| 19 | Stem lineage node of Mactroidea (*Mulinoides*-crown split): *Spisula*, younger than *Mulinoides* | *Spisula* | 61.6 | 59.2 | Conflict. Left undated and omitting *Spisula* from dating of other nodes because its sister genera are much younger than its oldest known member, the Late Paleocene *S. chulgunensis* (Krishtofovich 1947), Thanetian Khulgunskaya Suite, Kamchatka (see Gladenkov et al., 1997; Kafanov & Ogasawara, 2004; they put other species in *Spisula* that Moore, 2003 puts in *Mactomeris?*, so affinities of this species are uncertain). Younger is the earliest Eocene *S.* *parilis bistriata* (Harris, 1897), Lower Ypresian (NP10) Bashi Member of Hatchitigbee Formation, Ozark, Dale Co., Alabama (Palmer & Brann, 1965), and Lower Ypresian Sabinetown Formation, Sabinetown Bluff, Sabine Co., Alabama (Palmer & Brann, 1965). The types of *S. parilis bistriata* (held at the Paleontological Research Institution) are impossible to confirm as *Spisula* or *Mactromeris*, as the hinge is not visible and the shell is broken (J. Signorelli, personal observations); Krishtofovich’s 1947 species has not been re-figured or redescribed and is similarly difficult to place. Both *S.* *parilis bistriata* and *S. chulgunensis* require further evaluation given the conflict with molecular data.  The Paleocene species *Notospisula ewekoroensis* Adegoke, 1977 was described in a genus synonymized with *Spisula s.s.* by Beu (2004) and Huber (2010, 2015), but considered distinct by Signorelli (2023). The extant type species of *Notospisula* requires sequencing, and the fossil *N. ewekoroensis* requires further evaluation, as its description and figures do not convincingly fit this genus*,* which otherwise ranges Pliocene-Recent. Many other Eocene species have been assigned to *Spisula*, including two from the Lower Bartonian Gosport Formation of the U.S. Gulf Coast (Palmer & Brann, 1965): *S. parilis s.s.* (Conrad, 1833), which bears a striking resemblance to the extant *S. williamsi*, and *S. decisa* (Conrad, 1833), which shares shell outline features with *Mactrotoma* and *Simomactra* and whose placement in the genus is uncertain. Several other fossil taxa placed in *Spisula*, including *S.* *compressa* (Deshayes, 1832) and *S. semisulcata* (Lamarck, 1805) from the Lutetian of the Paris Basin and Hampshire Basin, have also been assigned to the Australian genus *Austromactra* (e.g. Pacaud & Le Renard, 1995), but may be better placed in *Eomactra*, as in Vincent (1929) and Tracey et al. (1996). |
| 20 | Crown Mactroidea | *Mactra* | 28.1 | 23.03 | Conflict. Left undated because oldest known *Spisula* is uncertain and because *Mactra* is younger than several genera that are more closely related to *Spisula*. It is also not certain if this is a bifurcation. *M. garreti* Ortmann, 1902, Upper Oligocene middle horizons of the Monte Leon Formation, Punta Quilla, Monte Entrada, Cañadon de los Misioneros, and Comandante Luis Piedra Buena, Argentina (del Rio, 2004). |
| 21 | *Mulinia*, younger than *Spisula* | *Mulinia* | 35.85 | 33.9 | Conflict. Left undated because oldest known *Spisula* is uncertain and because the topology can accommodate the relatively old age of *Mulinia* (see node 2) or *Spisula*, but not both. |
| 22 |  | *Anapella* | 3.6 | 2.59 | Conflict. Left undated because *Anapella* is younger than *Mactrinula*. *A. variabilis* (Tate, 1887), perhaps Upper Pliocene upper member of Dry Creek Sands, York Bore (Ludbrook, 1955) and Holden's Bore, 200 ft (Ludbrook, 1963:Fig. 4), South Australia (Ludbrook, 1955, age from Darragh, 2024; also Hallett Cove Sandstone, Oyster Banks at Blanche Point Cliffs, Aldinga Bay, South Australia (Ludbrook, 1955; Darragh, 2024); also *A. cycladea* (Lamarck, 1818), also Upper Pliocene upper member of Dry Creek Sands and Roe Calcarenite (Darragh, 2024). |
| 23 |  | *Raetella* | 23.03 | 20.44 | Conflict. Left undated because ages of *Raeta* and *Raetella* are mutually incompatible given the topology (both are older than *Meropesta* and *Anapella*). Tentatively, *Raetella* sp., Aquitanian Yoshigaura Formation = upper member of Taihu Group, Tsushima Islands, Japan (Masuda, 1970, pl. 6, fig. 7, as *Raeta* sp.; a small ovate shell with concentric plications, unlikely to be *Raeta s.s.*; age from Kimura et al., 1991). |
| 24 | *Mesodesma*, younger than *Paphies* | *Mesodesma* | 3.6 | 2.59 | Conflict. Left undated because *Mesodesma* is younger than *Donacilla*. *M. donacium* (Lamarck, 1818), Upper Pliocene Huacllaco Beds, southern Peru (DeVries, 2005; also Upper Pliocene Mactridenbanke, Horcon Formation, Horcon, central Chile (Tavera, 1960; Herm, 1969:68, 94, 119, accepted by Saul, 1989; Nielsen 2012 agrees on age of unit). |
| 25 | *Taria*, younger than *Paphies* | *Taria* | 3.6 | 2.59 | Conflict. Leaving undated because *Taria* is younger than *Donacilla*. “*Paphies*” n. sp. aff. *donacina* (Spengler, 1793; the type species of *Taria*) and “*Paphies*” n. sp. aff. *subtriangulata* (Wood, 1828; also put in *Taria* by Huber, 2010, 2015]), both Waipipian (=Lower Piacenzian) Mangere Shellbed, Otahuhu Formation, Māngere Wastewater Treatment Plant, New Zealand (Beu, 2019; Hayward et al., 2023). |

**Table S2. Potential oldest extant mactroids, not included in Table S1**

| **Taxon** | **Age max (Ma)** | **Age min (Ma)** | **Comments** |
| --- | --- | --- | --- |
| *Mactromeris* | 61.6 | 56 | Candidates include *M.*? *aequilateralis* (Waring 1917), Thanetian Martinez Formation, Simi Hills, Ventura Co, CA (Moore 2003, her query), and Thanetian Kachitka Formation, Kamchatka, Russia (Gladenkov et al. 1997), and *M. capayana* (Vokes 1939), Lower Ypresian Cerros Shale Member of Lodo Formation, Fresno Co, CA (Moore 2003; age from Groves & Squires 2021:7-8) and *M. tejonensis* (Packard 1916), Ypresian Meganos Formation (see Clark & Woodford 1927:105 for details), CA (Moore 2003). Younger is *M. ramonensis* (Packard 1916), Lower Priabonian-Lower Rupelian San Lorenzo Formation, Contra Costa Co, CA (Moore 2003, refined age from Baden et al. 2024) and Upper Priabonian-Lower Rupelian San Emigdio Formation, Kern Co., CA (Moore 2003). |
| *Oxyperas* | 51.9 | 47.8 | *O. flemingi* (Marwick 1960), Upper Ypresian Kauru Formation, Otaio Gorge, New Zealand (Beu & Maxwell 1990:98-99, 148, Maxwell 1978, who gives details on all known species; age for this locality from Beu & Marshall 2011:19). |
| *Crassula* | 44.5 | 41.2 | *C*. n.sp., Bortonian (=Upper Lutetian-Lower Bartonian, Raine et al. 2015), upper Ashburton River, Canterbury, NZ (Beu 2004, 2019). |
| *Paphies* | 39.5 | 35.85 | *P.* n.sp., Kaiatan (Upper Bartonian-Lower Priabonian, Raine et al. 2015), Waihaorunga, NZ (Beu 2019). |

**References for Tables S1 and S2**

Adegoke, O.S., 1969. Stratigraphy and paleontology of the marine Neogene formations in the Coalinga region. *University of California Publications in Geological Sciences* 80: 240 p.

Adegoke, O.S., 1977. Stratigraphy and paleontology of the Ewekoro Formation (Paleocene) of southwestern Nigeria. *Bulletins of American Paleontology* 71:1-379.

Alvinerie, J., Caralp, M., Anglada, R. and Catzigras, F., 1977. *Stratotype et parastratotype de l'Aquitanien*. Paris: Centre National de la Recherche Scientifique, 105 p.

Amano, K., 2024. Speciation, extinction and survival of molluscan species in the marginal sea–Plio-Pleistocene Omma-Manganji fauna in the Japan Sea borderland. *Bulletin of the Mizunami Fossil Museum* 51: 75-109.

Amano, K., Vermeij, G.J., and Narita, K., 1993. Early evolution and distribution of the gastropod genus *Nucella*, with special reference to Miocene species from Japan. *Transactions and Proceedings of the Palaeontological Society of Japan* 171: 237-248.

Amenábar, C.R., Guerstein, G.R., Alperin, M.I., Palma, E.D., Casadío, S., Belgaburo, A. and Rodríguez Raising, M., 2022. Eocene palaeoenvironments and palaeoceanography of areas adjacent to the Drake Passage: insights from dinoflagellate cyst analysis. *Palaeontology* 65: e12601.

Ayoub-Hannaa, W.S., Bengtson, P., Fürsich, F.T., and Andrade, E.J., 2019. Cenomanian-Turonian (Cretaceous) heterodont bivalves from the Sergipe Basin, Brazil. *Revue de Paléobiologie, Genève* 38: 151-228.

Baden, C.W., Shuster, D.L., Hourigan, J.H., Gooley, J.T., Cahill, M.R. and Hilley, G.E., 2024. Crustal block-controlled contrasts in deformation, uplift, and exhumation in the Santa Cruz Mountains, California, USA, imaged through apatite (U-Th)/He thermochronology and 3-D geological modeling. *Geological Society of America Bulletin* 136: 2789-2814.

Basterot, M. de, 1825. Mémoire géologique sur les environs de Bordeaux. Première partie comprenant les observations générales sur les mollusques fossiles, et la description particulière de ceux qu’on rencontre dans ce bassin. *Mémoires de la Société d’Histoire Naturelle de Paris* 2: 1-100.

Beu, A.G., 2004. Marine Mollusca of oxygen isotope stages of the last 2 million years in New Zealand. Part 1: Revised generic positions and recognition of warm-water and cool-water migrants. *Journal of the Royal Society of New Zealand* 34: 111-265.

Beu, A.G., 2006. Marine Mollusca of oxygen isotope stages of the last 2 million years in New Zealand. Part 2. Biostratigraphically useful and new Pliocene to Recent bivalves. *Journal of the Royal Society of New Zealand* 36: 151-338.

Beu, A.G., 2009. Before the ice: biogeography of Antarctic Paleogene molluscan faunas. *Palaeogeography, Palaeoclimatology, Palaeoecology* 284: 191-226.

Beu A.G., 2019. Catalogue of New Zealand Cenozoic marine Mollusca. Pers. comm. to DJ, December 2019; posthumous manuscript now being completed and prepared for publication by Henry Gard, GNS Science, Lower Hutt, New Zealand.

Beu, A.G., and Marshall, B.A., 2011. New Cenozoic records of genera and families from New Zealand (Mollusca, Gastropoda): Highlights from Phillip Maxwell’s collection. *New Zealand Journal of Geology and Geophysics* 54: 13-34.

Beu, A.G., and Maxwell, P.A., 1990. Cenozoic Mollusca of New Zealand. *New Zealand Geological Survey Paleontological Bulletin* 58: 518 p.

Campbell, L.D., 1993. Pliocene mollusks from the Yorktown and Chowan River Formations in Virginia. *Virginia Division of Mineral Resources Publication* 127: 259 p.

Charnelli, M., Gouiric Cavalli, S., Reguero, M.A. and Cione, A.L., 2024. Middle Eocene chondrichthyan fauna from Antarctic Peninsula housed in the Museo de la Plata, Argentina. *Advances in Polar Science* 35: 14-47.

Charo, M.P., Gordillo, S., Fucks, E.E., and Giaconi, L.M., 2014. Late Quaternary molluscs from the northern San Matías Gulf (Northern Patagonia, Argentina), southwestern Atlantic: faunistic changes and paleoenvironmental interpretation. *Quaternary International* 352: 26-47.

Chung, C.-T., and Kanno, S., 1975. Tertiary formations and their molluscan fauna in the Central Range and Foothills areas of northern Taiwan. *Mining Research and Service Organization, Taiwan, Report* 144: 32 p.

Clark, B.L., 1918. The San Lorenzo Series of middle California. A stratigraphic and palaeontological study of the San Lorenzo Oligocene Series of the general region of Mount Diablo, California. *University of California Publications, Bulletin of the Department of Geology* 11: 45-234.

Clark, B.L., and Woodford, A.O., 1927. The geology and paleontology of the type section of the Meganos formation (Lower Middle Eocene) of California. *University of California Publications in Geological Sciences* 17: 63-142.

Conrad, T.A., 1833. On some new fossil and Recent shells of the United States. *American Journal of Science and Arts* 23: 339-346.

Cossmann, M., 1908. Pélécypodes du Montien de Belgique. *Mémoires du Musée Royal d’Histoire Naturelle de Belgique* 5 (3), *Mémoire* 19: 1-76.

Cox, L.R., 1939. Miocene, Pliocene and Post-pliocene Mollusca from Mozambique. *Boletim dos Servicos de Industria, Minas Geologia, Serie de Geologia e Minas, Memorias e Communicacoes* 3: 1-39.

Crame, J.A., 1984. Neogene and Quaternary Mollusca from the Makran Coast, Pakistan. In: B.U. Haq and J.D. Milliman, eds., *Marine Geology and Oceanography of Arabian Sea and Coastal Pakistan.* Van Nostrand Reinhold, New York, pp.45-61.

Dall, W.H., 1890-1903. Contributions to the Tertiary fauna of Florida, with especial reference to the Miocene silex-beds of Tampa and the Pliocene beds of the Calooshatchie River. *Wagner Free Institute of Science of Philadelphia, Transactions* 3: 1-1654.

Dartevelle, E., and Freneix, S., 1957. Mollusques fossiles du Crétacé de la Côte occidentale d'Afrique du Cameroun à l'Angola. II. - Lamellibranches. *Annales du Musée Royal du Congo Belge, Série in-8, Sciences Géologiques* 20: 1-271.

Darragh T.A., 2024. A checklist of Australian marine Cenozoic Mollusca. *Memoirs of Museum Victoria* 83: 37-206.

del Rio, C.J., 2004. Tertiary marine molluscan assemblages of eastern Patagonia (Argentina): A biostratigraphic analysis. *Journal of Paleontology* 78: 1097-1022.

Deshayes, G.P., 1855. Descriptions of new shells from the collection of Hugh Cuming, Esq. *Proceedings of the Zoological Society of London* 22: 317-371.

DeVries, T.J., 2005. *Pterorytis pacanana* new species (Gastropoda: Muricidae): Circumstantial evidence for late Pliocene El Niño events in southern Peru. *Nautilus* 119: 164-168.

Dutton, A.L., Lohmann, K.C., and Zinsmeister, W.J., 2002. Stable isotope and minor element proxies for Eocene climate of Seymour Island, Antarctica. *Paleoceanography* 17: 1016.

Eames, F.E., 1970. Some thoughts on the Neogene/Palaeogene boundary. *Palaeogeography, Palaeoecology, Palaeoclimatology* 8:37-48.

Eames, F.E., and Clarke, W.J., 1967. Mayer’s stratotype area Aquitanian faunas. *Eclogae Geologicae Helvetiae* 60: 553-566.

Freneix, S., 1959. Mollusques fossiles du Crétacé de la Côte occidentale d’Afrique du Cameroun à l’Angola. III.-Conclusions stratigraphiques et paléontologiques. *Annales du Musée Royal du Congo Belge, Tervuren, Série in-8e, Sciences Géologiques* 24: 1-126.

Gardner, J.A., 1926-1928. The Molluscan fauna of the Alum Bluff Group of Florida, Parts I-V. *U.S. Geological Survey Professional Paper* 142-A-D: 249 p.

Gladenkov, Yu B., ed., 1997. *Lower Paleogene of West Kamchatka (stratigraphy, paleogeography, geological events)*. Moscow: GEOS Publishers, 367p. (in Russian).

Glenn, L.C., 1904. Systematic paleontology, Miocene Pelecypoda. Baltimore: Maryland Geological Survey, Miocene Volume, 274-401.

Glibert, M. and van de Poel, L., 1970. Les bivalvia fossiles du cénozoïque étranger des collections de l’Institut Royal des Sciences Naturelles de Belgique. VI (fin). Oligodontina: 2. Astartedontina et Septibranchida. *Mémoires de l'Institut Royal des Sciences Naturelles de Belgique* 84: 185 p.

Gmelin, J.F., 1791. Vermes. In: J.F. Gmelin, ed., Caroli a Linnaei … *Systema Naturae per Regna Tria Naturae,* Ed. 13. Tome 1(6). Lipsiae [Leipzig]: G.E. Beer, 3021-3909.

Groves, L.T., and Squires, R.L., 2021. Checklist of California Paleogene–Neogene marine Mollusca since Keen and Bentson (1944). *PaleoBios* 38: 360 p.

Hall, C.A., Jr., 2002. Nearshore marine paleoclimatic regions, increasing zoogeographic provinciality, molluscan extinctions, and paleoshorelines, California: Late Oligocene (27 Ma) to late Pliocene (2.5 Ma). *Geological Society of America Special Paper* 357: 1-489.

Harris, G.D., 1897. The Lignitic stage, Part I, stratigraphy and Pelecypoda. *Bulletins of American Paleontology* 2(9): 102 p.

Harzhauser, M., and Mandic, O., 2001. Late Oligocene gastropods and bivalves from the Lower and Upper Austrian Molasse Basin. In: W.E. Pillar and M.W. Rasser, eds., Paleogene of the Eastern Alps. *Österreichische Akademie der Wissenschaften, Schriftenreihe der Erdwissenschaftlichen Kommissionen* 14: 671-795.

Hatai, K.M., 1940. On some fossils from the Ninohe District, Mutu Province, northeast Honsyû, Japan (No. 1). *Bulletin of the Biogeographical Society of Japan* 10: 119-138,

Hayward, B.W., Stolberger, T.F., Collins, N., Beu, A.G. and Blom, W., 2023. A diverse Late Pliocene fossil fauna and its paleoenvironment at Māngere, Auckland, New Zealand. *New Zealand Journal of Geology and Geophysics* 67: 551-572.

Herm, D., 1969. Marines Pliozän und Pleistozän in Nord- und Mittel-Chile unter besonderer Berücksichtigung der Entwicklung der Mollusken-Faunen. *Zitteliana* 2: 159 p.

Higo, S., Callomon, P. and Goto, Y., 1999. *Catalogue and bibliography of the marine shell-bearing Mollusca of Japan*. Osaka: Elle Scientiﬁc Publications, 749 p.

Hölzl, O., 1962. Die Molluskenfauna der oberbayrischen marinen Oligocänmolasse zwischen Isar und Inn und ihre stratigraphische Auswertung. *Geologica Bavarica* 50: 1-143.

Huber, M., 2010. *Compendium of bivalves*. Harxheim, Germany: ConchBooks.

Huber, M., 2015. *Compendium of Bivalves 2. A full-color guide to the remaining seven families.* Hackenheim, Germany: ConchBooks.

Itoh, Y., and Watanabe, M., 2000. Refined magnetostratigraphy of the Early Miocene sequence in the Yatsuo area, central Japan. *Bulletin of the Geological Survey of Japan* 51: 37-45.

Ivany, L.C., Lohmann, K.C., Hasiuk, F., Blake, D.B., Glass, A., Aronson, R.B., and Moody, R.M., 2008. Eocene climate record of a high southern latitude continental shelf: Seymour Island, Antarctica. *Geological Society of America Bulletin* 120: 659-678.

Kafanov, A.I., 1991. *Shelf and continental slope bivalve molluscs of the Northern Pacific Ocean: a check-list.* Vladivostok: Far Eastern Branch of the Academy of Sciences of the USSR.

Kafanov, A.I., and Ogasawara, K., 2004. Neogene and Paleogene Molluscan (Bivalvia) Cenozones of the Russian Northeast. *Science Reports of the Institute of Geoscience, University of Tsukuba* B25: 9-45.

Kanno, S., 1958. New Tertiary molluscs from the Chichibu Basin, Saitama Prefecture, central Japan. *Science Reports of the Tokyo Kyoiku Daigaku, Section C, Geology, Mineralogy and Geography* 55: 157-229.

Kanno, S., and Chung, C.-T., 1973. Molluscan fauna from the so-called Paleogene formations in northern Taiwan. *Contributions to the Geology and Palaeontology of Southeast Asia* 13: 91-127.

Kimura, T., Hayami, I., and Yoshida, S., 1991. *Geology of Japan*. Tokyo: University of Tokyo Press.

Krishtofovich, L.V., 1947. Stratigraphy and fauna of the Tighil Series of the western

coast of Kamchatka. *Trudy Vsesoyuznogo Neftyanogo Nauchno-Issledovatel’skogo*

*Geologorazvedochnogo Instituta (VNIGRI), Novaya Seriya (Transactions of the All-Union Petroleum Geological-Prospecting Institute, New Series*) 23:1–151 (in Russian).

Lamprell, K., and Whitehead, T., 1992. Bivalves of Australia. Crawford House Press, Bathurst, New South Wales, 182 p.

Lesport, J.F., and Cahuzac, B., 2005. Découverte des sables à Mactres de la base du Burdigalien à Martillac (Nord Aquitaine; SW France); données paléontologiques. *Annales de Paléontologie* 91: 73-116.

Licht, A., Reisberg, L., France-Lanord, C., Soe, A.N., and Jaeger, J.-J., 2014. Cenozoic evolution of the central Myanmar drainage system: Insights from sediment provenance in the Minbu Sub-Basin*. Basin Research* 28: 237-25.

Ludbrook, N.H., 1955. The molluscan fauna of the Pliocene strata underlying the Adelaide Plains. Part II. Pelecypoda. *Transactions of the Royal Society of South Australia* 77: 42-64.

Ludbrook, N.H., 1963. Correlation of the Tertiary rocks of South Australia. *Transactions of the Royal Society of South Australia* 87: 5-15.

Lutaenko, K.A., and Héros, V., 2011. Type material of bivalve mollusks collected during *Vénus* Voyage (1836–1839) from Kamchatka, Russia, in the Muséum National d'Histoire Naturelle, Paris. *Malacologia* 54: 77-85.

Marasti, R., 1991. Bivalvi polisiringi del Pliocene, viventi nel Mediterraneo: Distribuzione e osservazioni. II. *Acta naturalia de l'Ateneo parmense* 27: 39-63.

Marshall, B.A., and Spencer, H.G., 2013. Comments on some taxonomic changes affecting marine Bivalvia of the New Zealand region recently introduced in *Huber’s Compendium of bivalves*, with some additional taxonomic changes. *Molluscan Research* 33:40-49.

Marwick, J., 1960. Early Tertiary Mollusca from Otaio Gorge, South Canterbury. *New Zealand Geological Survey Paleontological Bulletin* 33: 32 p.

Masuda, K., 1970. Molluscan fauna from the Taishu Group, Tsushima Islands, Nagasaki Prefecture, Japan. *Memoirs of the National Science Museum Tokyo* 3: 25-32.

Masuda, K., and Huang, C.-Y., 1990a. Check list of Tertiary and Quaternary Molluscan in Taiwan. *Acta Geologica Taiwanica* 28: 31-76.

Masuda, K., and Huang, C.-Y., 1990b. Miocene Pelecypoda in the western foothills of northern Taiwan (Part 2: Systematic description). *Bulletin of the National Museum of Natural Sciences, Taiwan* 2: 141-189.

Matsubara, T., 1997. Redescription of two fossil mactrids (Mollusca: Bivalvia) from the Lower Miocene of northeast Japan with discussion of their taxonomical status. *Saito Ho-on Kai Museum of Natural History Research Bulletin* 65: 13-25.

Maxwell, P.A., 1978. Taxonomic and nomenclatural notes on some New Zealand Cenozoic Mollusca, with descriptions of new taxa. *New Zealand Journal of Zoology* 5: 15-46.

Mayer[-Eymar], C., 1867. Catalogue systématique et descriptive des Mollusques tertiaires du Musée fédéral de Zurich. II. Mactrides et Pholadomyides. *Vierteljahrsschrift der Naturforschenden Gesellschaft in Zurich* 12: 241-303.

Moore, E.J., 2003, Tertiary marine pelecypods of California and Baja California, Chapter F: Crassatellidae, Cardiidae, Mactridae, Mesodesmatidae, Solenidae, Pharidae: http://www.cmug.com/~chintimp/Tertiary.pelecypods.htm, 107 p., 14 pl.

Mourier, T., Bengtson, P., Bonhomme, M., Buge, E., Cappetta, H., Crochet, J.Y., Feist, M., Hirsch, K., Jaillard, E., Laubacher, G., and Lefranc, J.P., 1988. The Upper Cretaceous-Lower Tertiary marine to continental transition in the Bagua basin, northern Perú. *Newsletters on Stratigraphy* 19: 143-177.

Nielsen, S.N., 2012. What is "typical" Pliocene? An evaluation of new and long known Chilean Pliocene mollusk faunas. *13 Congreso Geológico Chileno, Antofagasta, Chile*, 728-730.

Okutani, T., 2000. *Marine mollusks in Japan*. Tokai University Press, 1173 p.

Oliver, P.G., 1992. *Bivalved seashells of the Red Sea*. Wiesbaden: Hemmen; Cardiff: National Museum of Wales, 330 p.

Olsson, A.A., 1944. Contributions to the paleontology of northern Peru. Part VII. The Cretaceous of the Paita region. *Bulletins of American Paleontology* 28: 163-305.

Oostingh, C.H., 1931. Beitrag zur Kenntnis der Molluskenfauna von Süd-Sumatra. *Archiv für Molluskenkunde* 63: 166-255.

Oostingh, C.H., 1935. Mollusken des Pliozäns von Boemiajoe (Java). *Dienst van den mijnbouw in Nederlandsch-Indië, Wetenschappelijke Mededeelingen* 26: 1-247.

Ortmann, A., 1902. Tertiary invertebrates. In: W.B. Scott, ed., *Reports of the Princeton University Expedition to Patagonia 1896-1899, Paleontology I, Part 2, vol. 4*. Princeton, New Jersey: J. Pierpoint Morgan Publishing Foundation, 45-325.

Oyama, K., 1973. Revision of Matajiro Yokoyama’s type Mollusca from the Tertiary and Quaternary of the Kanto area. *Palaeontological Society of Japan Special Paper* 17.

Packard, E.L., 1916, Mesozoic and Cenozoic Mactrinae of the Pacific Coast of North America. *California University Publications, Department of Geology Bulletin* 9: 261–360.

Pacaud, J.-M., and Le Renard, J., 1995. Révision des Mollusques paléogènes du bassin de Paris. IV – Liste systématique actualisée. *Cossmanniana* 3: 151-187.

Palmer, K.V.W., and Brann, D.C., 1965. Catalogue of the Paleocene and Eocene Mollusca of the southern and eastern United States. Part I. Pelecypoda, Amphineura, Pteropoda, Scaphopoda, and Cephalopoda. *Bulletins of American Paleontology* 48: 1-466.

Paulay, G., 1996. Dynamic clams: changes in the bivalve fauna of Pacific islands. *American Malacological Bulletin* 12: 45-57.

Petuch, E.J., Drolshagen, M., 2010. *Molluscan paleontology of the Chesapeake Miocene*. Boca Raton: CRC Press, Boca Raton.

Powell, A.W.B., and Bartrum, J.A., 1929. The Tertiary (Waitematan) molluscan fauna of Oneroa, Waiheke Island. *Transactions and Proceedings of the New Zealand Institute*. 60: 395–447.

Powell, C.L., II, and Rugh, N.S., 2025. New species of the deep-water bivalve genera *Acesta* (Limidae, Bivalvia, Mollusca) and a questionable *Malletia* (Malletiidae, Bivalvia, Mollusca) from the Eocene of southern California. *PaleoBios* 42 (5): 1-13.

Ridd, M.F., and Racey, A., 2015. Onshore petroleum geology of Myanmar: Central Burma Depression. In: Racey, A., and Ridd, M.F. (eds.), *Petroleum Geology of Myanmar*. Geological Society of London Memoir 45: 21-50.

Raine, J.I., Beu, A.G., Boyes, A.F., Campbell, H.J., Cooper, R.A., Crampton, J.S., Crundwell, M.P., Hollis, C.J., and Morgans, H.E.G., 2015. Revised calibration of the New Zealand Geological Timescale. *GNS Science Report* 2012/39: 53 p.

Reeve, L.A., 1844. Monograph of the genus Arca. *Conchologia Iconica* vol. 2, pl. 1-17 and unpaginated text. London: L. Reeve & Co. [pl. 1-2: December 1843; 3-4: January 1844; 5-8: February 1844; 9-12: March 1844; 13-14: April 1844; 15-16: May 1844; 17: June 1844].

Saul, L.R., 1989. California Late Cretaceous donaciform bivalves. *Veliger* 32: 188-208.

Scarlato, O.A., 1981. Bivalve mollusks of temperate waters of the northwestern Pacific. *Opredeliteli po Faune SSSR* 126: 1–480. [In Russian]

Schultz, O., 2003. Bivalvia neogenica (Lucinoidea-Mactroidea). *Catalogus Fossilium Austriae* 1: 381-690.

Sepkoski, J.J., Jr., 2002. A compendium of fossil marine animal genera. *Bulletins of American Paleontology* 363: 560 p.

Signorelli, J.H., 2019. *The Superfamily* Mactroidea *(*Mollusca: Bivalvia*) in American waters: An illustrated catalogue of Recent species*. Cham: Springer Nature Switzerland, 151 p.

Signorelli, J.H., 2023. Catalogue of fossil genera of Mactridae (Mollusca: Bivalvia). *Journal of Paleontology* 97: 823-852.

Stilwell, J.D., and Zinsmeister, W.J., 1992. Molluscan systematics and biostratigraphy, lower Tertiary La Meseta Formation, Seymour Island, Antarctic Peninsula. *American Geophysical Union, Antarctic Research Series* 55: 192 p.

Studencka, B., Gontsharova, I. A., and Popov, S.V., 1998. The bivalve faunas as a basis for reconstruction of the Middle Miocene history of the Paratethys. *Acta Geologica Polonica* 48: 285-342.

Sutherland, F.L. and Kershaw, R.C., 1971. The Cainozoic geology of Flinders Island, Bass Strait. *Papers and Proceedings of the Royal Society of Tasmania* 105: 151–175.

Tate, R., 1887. The lamellibranchs of the Older Tertiary of Australia. (Part II.) *Transactions of the Royal Society of South Australia* 9: 142-189.

Tavera J., 1960. El Plioceno de Bahía Horcón en la provincia de Valparaíso. *Facultad de Ciencias Físicas y Matemáticas, Universidad de Chile, Anales* 17: 346-367.

Tracey, S., Todd, J.A., Le Renard, J., King, C., and Goodchild, M., 1996. Distribution of Mollusca in Units S1 to S9 of the Selsey Formation (middle Lutetian), Selsey Peninsula, West Sussex. *Tertiary Research* 16: 97-139.

Valenzuela-Toro, A.M., Pyenson, N.D., Costa, D.P., Clementz, M., and Koch, P.L., 2024. Stable isotope evidence for resource partitioning in extinct marine carnivores. *Palaeogeography, Palaeoclimatology, Palaeoecology* 649: 112302.

Van Urk, R.M., 1980. Fossil and Recent *Lutraria* (Mollusca, Bivalvia) in Europe, with descriptions of four new species. *Mededelingen van de Werkgroep voor tertiaire en kwartaire Geologie* 17: 235-266.

Vincent, E., 1929. La Mactre et l'Herouvalie des sables de Wemmel. *Annales de la Société Royale Zoologique de Belgique* 59 (for 1928): 55-58.

Vokes, H.E., 1939. Molluscan faunas of the Domengine and Arroyo Hondo formations of the California Eocene. *Annals of the New York Academy of Sciences* 38:1–246.

Vongpanich, V., 2000. Family Mactridae (Mollusca: Bivalvia) in Thai waters. *Phuket Marine Biological Center Special Publication* 21: 483-498.

Ward, L.W., 1992. Molluscan biostratigraphy of the Miocene Middle Atlantic Coastal Plain of North America. *Virginia Museum of Natural History* 2: 159p.

Waring, C.A., 1917. Stratigraphic and faunal relations of the Martinez to the Chico and Tejon of southern California. *Proceedings of the California Academy of Sciences Series* 4, 7: 41-124.

Woodring, W.P., Stewart, R., and Richards, R.W., 1940. Geology of the Kettleman Hills oil field, California: Stratigraphy, paleontology, and structure. *U.S. Geological Survey Professional Paper* 195: 170 p.

Zimmt, J.B., Kidwell, S.M., Lockwood, R., and Thirlwall, M., 2022. Strontium isotope stratigraphy reveals 100 ky-scale condensation, beveling, and lateral shingling of transgressive shell beds in the Maryland Miocene. *Palaios* 37: 553-573.
